# Drivers of methane-cycling archaeal abundances, community structure, and catabolic pathways in continental margin sediments

**DOI:** 10.3389/fmicb.2025.1550762

**Published:** 2025-02-06

**Authors:** Longhui Deng, Damian Bölsterli, Clemens Glombitza, Bo Barker Jørgensen, Hans Røy, Mark Alexander Lever

**Affiliations:** ^1^Institute of Biogeochemistry and Pollutant Dynamics, Swiss Federal Institute of Technology, ETH Zurich, Zurich, Switzerland; ^2^School of Oceanography, Shanghai Jiao Tong University, Shanghai, China; ^3^Center for Geomicrobiology, Department of Bioscience, Aarhus University, Aarhus, Denmark; ^4^Marine Science Institute, University of Texas at Austin, Port Aransas, TX, United States

**Keywords:** methanogens, ANMEs, methanogenesis, Anaerobic Oxidation of Methane (AOM), Gibbs energy, stable isotopes, redox gradients, anoxic marine sediment

## Abstract

Marine sediments contain Earth’s largest reservoir of methane, with most of this methane being produced and consumed *in situ* by methane-cycling archaea. While numerous studies have investigated communities of methane-cycling archaea in hydrocarbon seeps and sulfate–methane transition zones, less is known about how these archaea change from the seafloor downward throughout diffusion-dominated marine sediments. Focusing on four continental margin sites of the North Sea-Baltic Sea transition, we here investigate the *in situ* drivers of methane-cycling archaeal community structure and metabolism based on geochemical and stable carbon-isotopic gradients, functional gene (*mcr*A) copy numbers and phylogenetic compositions, and thermodynamic calculations. We observe major changes in community structure that largely follow vertical gradients in sulfate concentrations and lateral gradients in organic carbon reactivity and content. While methane-cycling archaeal communities in bioturbated and sulfatic zones are dominated by known methyl-disproportionating *Methanosarcinaceae* and putatively CO_2_-reducing *Methanomicrobiaceae*, the communities change toward dominance of methane-oxidizing taxa (ANME-2a-b, ANME-2c, ANME-1a-b) in sulfate–methane transition zones (SMTZs). By contrast, the underlying methanogenesis zones are dominated by the physiologically uncharacterized ANME-1d, new genus-level groups of putatively CO_2_-reducing *Methanomicrobiaceae*, and methyl-reducing *Methanomassiliicoccales*. Notably, *mcr*A copy numbers of several major taxa increase by 2 to 4 orders of magnitude from the sulfatic zone into the SMTZ or methanic zone, providing evidence of net population growth in subsurface sediment. We propose that burial-related geochemical changes cause methane-cycling archaea in continental margin sediments to go through three successional stages (sulfatic, SMTZ, methanic). Herein, the onset of each new successional stage is characterized by a period of growth- and mortality-driven turnover in the dominant taxa.

## Introduction

Despite being Earth’s largest methane reservoir, marine sediments are only minor sources of atmospheric methane compared to freshwater sediments (Reeburgh, 2007). High concentrations of sulfate in seawater restrict most microbial methane production to deeper sediment layers beneath the ‘sulfatic zone’ (Jørgensen, 2021) and promote the anaerobic oxidation of >90% of marine sedimentary methane before it can reach the seafloor or overlying water (Reeburgh, 2007). Nonetheless, recent studies suggest that methane emissions from marine sediments are higher than previously thought, particularly in coastal and continental shelf environments (Weber et al., 2019; Lapham et al., 2024). These emissions may increase in the future due to eutrophication and climatic warming (James et al., 2016), which promote bottom water oxygen depletion and water column stratification and lead to shallowing of methanic zones (Dean et al., 2018; Bianchi et al., 2021).

Most sedimentary methane is produced by methanogenic archaea (methanogens). The latter convert microbial fermentation products to methane via a process known as methanogenesis (Schink, 1997). The distribution of methanogens is partially controlled by competition with respiring microorganisms that use oxygen, nitrate, metal oxides (Mn(IV), Fe(III)), or sulfate as electron acceptors. These organisms typically have higher energy gains from the same energy substrates than methanogens (Lovley and Goodwin, 1988). As a result, methanogenesis often dominates respiration only in deeper, so-called ‘methanic zones’ (Jørgensen, 2021), in which these energetically superior electron acceptors are depleted (Jørgensen and Kasten, 2006). A major fraction of the methane produced in methanic zones diffuses into overlying sulfate–methane transition zones (SMTZs), where it is consumed by Anaerobic Oxidation of Methane (AOM), a process that is performed by ANaerobic MEthanotrophic archaea (ANME), which are closely related to methanogenic archaea. These ANMEs, in many cases through syntrophic associations with bacteria, couple AOM to the reduction of sulfate (Boetius et al., 2000), metal oxides (Ettwig et al., 2016), or nitrate (Haroon et al., 2013). Among these, AOM coupled to sulfate reduction is by far the most important methanotrophic pathway in anoxic marine sediments (Egger et al., 2018).

Multiple archaeal taxa have been linked to methanogenesis and AOM in marine sediments. Previous studies suggest that the dominant methanogens belong to the euryarchaeal orders *Methanomicrobiales*, *Methanosarcinales*, *Methanocellales*, *Methanobacteriales* and *Methanomassiliicoccales* (Lever, 2013; Wen et al., 2017). Known ANMEs are also Euryarchaeota and include the order-level ANME-1, the family-level ANME-2a-b (“Candidatus Methanocomedenaceae”), ANME-2c (“Candidatus Methanogasteraceae”), and ANME-2d (*Methanoperedenaceae*) (order *Methanosarcinales*), and the genus-level ANME-3 (“Candidatus Methanovorans”) (family *Methanosarcinaceae*; Chadwick et al., 2022).

All known archaeal methanogens produce methane via the reductive acetyl CoA pathway, and reduce methyl coenzyme M to methane via methyl coenzyme M reductase as a terminal step (Liu and Whitman, 2008). Four variations of this pathway are known, which differ in carbon substrates: (a) CO_2_ reduction, typically involving H_2_ or formate as electron donors (‘hydrogenotrophic’); (b) acetate disproportionation (‘aceticlastic’); (c) methylated compound, e.g., methanol, methylamines, or methylsulfides, cycling by methyl group disproportionation or methyl group reduction with H_2_ (‘methylotrophic’; Whitman et al., 2014); and (d) O-demethylation of methoxylated aromatic compounds (methoxydotrophic; Mayumi et al., 2016). In addition, some methanogens, e.g., *Methanothrix* and *Methanosarcina* (both *Methanosarcinales*), perform CO_2_ reduction by extracellular electron transfer (EET) via conductive structures that connect to partner organisms, minerals or organic carbon compounds (Rotaru et al., 2014; Gao and Lu, 2021).

Most biogenic methane is believed to be produced via the aceticlastic and hydrogenotrophic reactions (Conrad, 1999, 2020), with CO_2_ reduction prevailing in methanic zones of marine sediments. This inference is mainly based on measurements indicating that CO_2_ reduction produces more negative carbon isotopic signatures (δ^13^C-CH_4_: −60 to −110‰) than aceticlastic methanogenesis (δ^13^C-CH_4_: −50 to −60‰; Whiticar et al., 1986), and by direct measurements with radiolabeled CO_2_ and acetate (Beulig et al., 2018). By contrast, methyl group disproportionation has been shown to frequently dominate methanogenic reactions in sulfate-reducing marine surface sediments (Xiao et al., 2018; Zhuang et al., 2018). This co-existence of methanogens with sulfate reducers at high sulfate concentrations has been attributed to methylated compounds being “non-competitive” substrates that are not used by most sulfate reducers (Oremland et al., 1982; King, 1984).

To date, most research on sedimentary methane-cycling archaea has focused on advective systems, such as hydrocarbon seeps (Knittel et al., 2005; Lloyd et al., 2010; Orcutt et al., 2010; Yanagawa et al., 2011; Ruff et al., 2015; Takano et al., 2018; Niu et al., 2022), and on SMTZs of diffusion-dominated sediments (Harrison et al., 2009; Beulig et al., 2019). Comparatively less is known about the community structure and pathways of methanogenesis and AOM in bioturbated surface sediments [bioturbation zone (BZ)], sulfatic zones (SZs), and methanic zones (MZs) of diffusion-dominated sediments, and how these communities and pathways respond to vertically changing geochemical conditions and laterally changing sedimentary settings. Here we explore the diversity, community structure and pathways of methane-cycling communities and their potential environmental drivers at four continental margin sites at the North Sea-Baltic Sea transition. We integrate chemical, stable isotopic, and Gibbs energy data with methane-cycling archaeal abundance and community data from three sites that range from coastal eutrophic to off-shore oligotrophic and differ greatly in sedimentation rates, organic carbon inputs, electron acceptor distributions, as well as microbial and macrofaunal activity and community structure. We used sediment cores previously described by Kristensen et al. (2018) and Deng et al. (2020), three of which extend well into the MZ and were sampled at high depth resolution across the SMTZ, thus allowing for detailed analyses of methanogenic and methanotrophic community shifts across this important biogeochemical transition. Based on our comprehensive geochemical and microbiological dataset, we identify key drivers of methanogenic and methanotrophic communities in continental margin sediments.

## Materials and methods

### Site description

**Figure 1:**
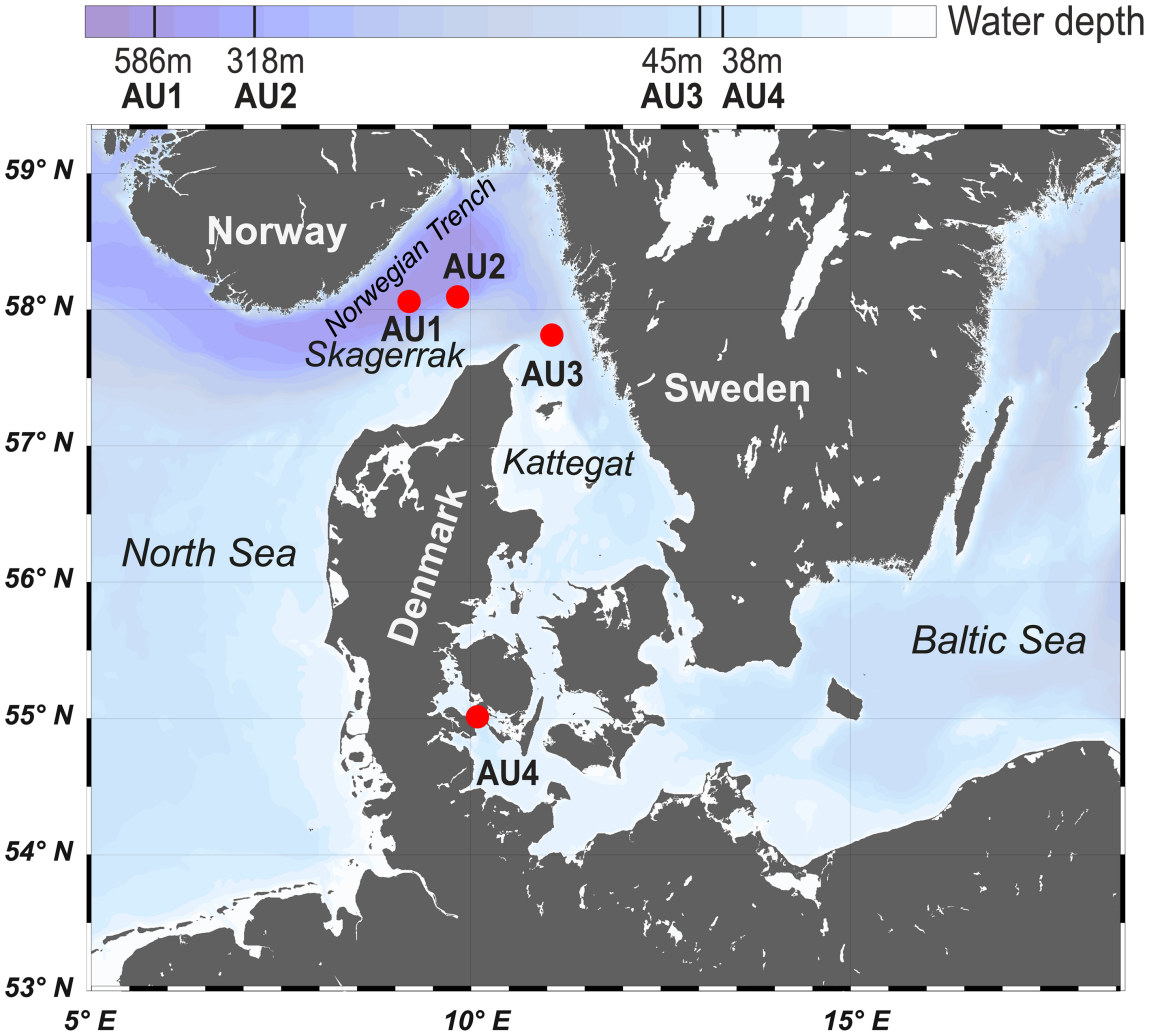
Map of the Skagerrak-Kattegat-Belt Sea area. The four sampling sites (AU1-AU4) fall along a gradient of decreasing water depth and are indicated by red dots.

All samples were taken during a cruise of the R/V *Aurora* in August–September 2014. The four sites AU1 (586 m water depth), AU2 (319 m), AU3 (43 m), and AU4 (37 m) are located along a water-depth and bioturbation gradient from the North Sea into the Baltic Sea (Figure 1). Organic matter reactivity and sedimentation rates (AU1: 0.14 cm yr^−1^; AU2: 0.27 cm yr^−1^; AU3: 0.30 cm yr^−1^; AU4: 0.33 cm yr^−1^) increase with decreasing water depth and distance to shore (Deng et al., 2020). AU1 and AU2 are in the Skagerrak region, with AU1 being located near the bottom of the Norwegian Trench and AU2 on the southern slope of the same trench. Both sites are characterized by silty clay and low-reactivity allochthonous organic matter and have high rates of iron and manganese reduction in the top 10 cm (Kristensen et al., 2018; Deng et al., 2020). AU3 in the northern Kattegat is dominated by fine sands, while AU4 in Lillebælt Strait, which leads into the Baltic Sea, is dominated by silty clay. AU4 is subject to seasonal bottom water hypoxia and was sulfidic with the exception of a 1 mm thick oxidized surface layer (Kristensen et al., 2018). Macrofaunal biomass increases from AU1 to AU3, but macrofauna was absent from AU4 at the time of sampling (Kristensen et al., 2018; Deng et al., 2020). The depths of macrofaunal ventilation and sediment reworking are lowest at AU1 (ventilation: 0–5 cm; reworking: 0–8 cm) and in a similar range at AU2 (ventilation: 0–13 cm; reworking: 0–40 cm) and AU3 (ventilation: 0–12 cm; reworking: 0–35 cm) (Deng et al., 2020).

### Sampling scheme

The top 40–50 cm of sediment was sampled using a Rumohr corer, a lightweight (45 kg) gravity corer without a core catcher, which can recover cores with undisturbed surface sediments. All deeper sediments [to 500 cm below seafloor (cmbsf)] were collected using a conventional gravity corer with a 6 m steel barrel, core catcher, internal PVC core liner, and approximately 1,000 kg of lead weight. Sediment porewater was sampled from Rumohr cores in 5 cm depth intervals. Porewater from gravity cores was collected in 10 cm intervals in the upper 1 m, and in 25 cm intervals below. Porewater was extracted using rhizon samplers (Rhizosphere, The Netherlands) that were inserted horizontally through 4 mm wide holes drilled into the side of the PVC core liners. Of the porewater samples, a 1 mL aliquot was immediately used for pH and alkalinity determination. In addition, 1 mL was preserved with 10 μL saturated HgCl_2_ at 4°C for DIC and δ^13^C-DIC analyses, 1 mL was stored at 4°C for dissolved SO_4_^2−^ quantification, and 2–4 mL were frozen at −20°C for analyses of volatile fatty acid (VFA) concentrations. Subsequently, the Rumohr cores were extruded in 2 cm intervals to 20 cm depth followed by 4 cm intervals to 48 cm depth, while the gravity cores were sampled at 8–10 cm depth intervals. All sediment for DNA, methane, δ^13^C-methane, total organic carbon (TOC), and δ^13^C-TOC analyses was sampled using 5 mL sterile, cut-off syringes. Samples for DNA, TOC, and δ^13^C-TOC analyses were immediately frozen at −20°C and transferred to −80°C upon arrival at the home laboratory. Samples for analyses of methane concentrations and δ^13^C-methane were preserved in saturated NaCl (6 M) and stored at 4°C until measurement.

### DNA extraction

DNA was extracted from sediments following lysis protocol II of Lever et al. (2015) using 0.2 g of wet sediment per sample. This protocol combines chemical (lysis solution I) and mechanical cell lysis (bead-beating: 0.1 mm Zirconium beads), 2× washing with chloroform:isoamyl alcohol (24:1), and precipitation with linear polyacrylamide, NaCl and ethanol. DNA was purified according to protocol A of the CleanAll DNA/RNA Clean-Up and Concentration Micro Kit (Norgen Biotek Corp., Canada). DNA extracts were the same as previously used for 16S rRNA gene quantification and sequencing in Deng et al. (2020).

### Quantitative PCR (qPCR)

*mcr*A gene copy concentrations in DNA extracts were quantified on a LightCycler 480 II (Roche Life Science, Switzerland) by qPCR assays using the Mlas_F (5′- GGT GGT GTM GGD TTC ACM CAR TA −3′) / McrA-rev (5′- CGT TCA TBG CGT AGT TVG GRT AGT −3′) primer pair (Steinberg and Regan, 2009) and the LightCycler 480 SYBR Green I Master reaction mix (Roche Life Science, Switzerland). Thermal cycler protocols consisted of (1) enzyme activation and initial denaturation (95°C, 5 min) followed by (2) 50 cycles of (a) denaturation (95°C, 10 s), (b) annealing (53°C, 20 s), (c) elongation (72°C, 30 s), and (d) fluorescence measurement (84°C, 5 s), and lastly (3) a stepwise melting curve from 95 to 53°C in 1 min intervals to check for primer specificity. Plasmids of *mcr*A from *Methanocorpusculum parvum* were applied as standards. All standards and samples were measured in duplicate.

### Sequencing and bioinformatics

*mcr*A amplicon libraries were prepared according to a published workflow that includes an initial booster PCR to increase amplicon copy numbers, followed by a “tailed primer PCR” to attach sequencing adaptors, and a final “index PCR” in which PCR products from each sample were labeled with sample-specific barcodes (Deng et al., 2020; Supplementary Information). Herein the number of PCR cycles was kept to a minimum to minimize primer biases. Throughout these PCR assays, we used the same primer pair and PCR conditions as for qPCR, except that we used the KAPA HiFI Hot Start ReadyMix instead of the SybrGreen I Master reagents. The *mcr*A amplicons were sequenced at ETH Zurich’s Centre for Genetic Diversity (https://gdc.ethz.ch/) using the Illumina MiSeq platform (Illumina Inc., California, USA). Raw reads were quality-checked by *FastQC*,^1^ read ends trimmed using *seqtk*,^2^ paired end reads merged using *flash* (Magoč and Salzberg, 2011), primer sites trimmed by *usearch* (Martin, 2011), and quality filtering done by *prinseq* (Schmieder and Edwards, 2011). Zero-radius Operational Taxonomic Units (ZOTUs) were generated using the UNOISE3 algorithm (Edgar, 2016) and clustered using a 97% identity threshold to generate 97% ZOTUs’ (referred to as ‘ZOTUs’ from now on). Taxonomic assignments were done in ARB^3^ using neighbor-joining phylogenetic trees with Jukes Cantor correction and 1,000 bootstrap replicate calculations. These trees were based on a public database (*mcrA4All*; Lever et al., 2023)^4^ with >2,400 high-quality, optimally aligned *mcr*A sequences from pure culture, amplicon sequencing, metagenome, and whole-genome studies. All *mcr*A ZOTU sequences are publicly accessible at the National Center for Biotechnology Information homepage (Accession #: KIEX00000000; BioProject: PRJNA1066864; BioSample: SAMN39507872).

### Geochemical analyses

Depth profiles of TOC and δ^13^C-TOC and porewater concentrations of sulfate, methane, and DIC were published previously (Marshall et al., 2019; Deng et al., 2020). δ^13^C-DIC and δ^13^C-methane were analyzed as outlined in Lapham et al. (2024; all values reported in reference to VPDB). Concentrations of volatile fatty acids (VFAs) were determined by 2D-ion chromatography as previously published (Glombitza et al., 2014). pH was measured immediately after pore water retrieval followed by acid titration to determine alkalinity. 0.5 mL of pore water sample were titrated with diluted HCl (20, 40, or 80 mM) to reach an end pH value between 3.5 and 3.9. Alkalinity was calculated from the start and endpoint pH and the added amount of acid according to a standard method (Grasshoff et al., 1983).

### Gibbs energy calculations

Gibbs energies (*ΔG_r_*) of (1) methanogenesis reactions from H_2_ + CO_2_ (HCO_3_^−^ + 4 H_2_ + H^+^ ➔ CH_4_ + 3 H_2_O), acetate (CH_3_COO^−^ + H_2_O ➔ CH_4_ + HCO_3_^−^), methanol (4 CH_3_OH ➔ 3 CH_4_ + HCO_3_^−^ + H_2_O + H^+^), and methanol+H_2_ (CH_3_OH + H_2_ ➔ CH_4_ + H_2_O), (2) anaerobic acetate oxidation (CH_3_COO^−^ + 4 H_2_O ➔ 2 HCO_3_^−^ + 4 H_2_ + H^+^), and (3) sulfate-dependent AOM (SO_4_^2−^ + CH_4_ ➔ HS^−^ + HCO_3_^−^ + H_2_O) were calculated based on the equation


$$\Delta G_{r}=\Delta G_{r}^{0}+\text{RTln}\;Q_{r}$$

where ΔG*_r_*^0^ is the Gibbs energy (kJ mol^−1^ of reaction) at standard concentrations (1 M per each reactant and product, pH 7.0), corrected for *in situ* temperature T (K) and pressure *p* (bar) based on standard enthalpies and molar volumes as outlined in Stumm and Morgan (1996), R is the universal gas constant (0.008314 kJ mol^−1^ K^−1^), and *Q_r_* the quotient of product and reactant activities. Calculations were done for measured concentrations of DIC, acetate, methane, and sulfate, and measured pH values. For H_2_, methanol, and hydrogen sulfide (HS^−^) concentrations, which were not measured, we performed calculations for concentrations that were estimated to be at the lower and upper *in situ* extremes of these chemicals (H_2_: 0.1 nM and 10 nM; methanol: 1 nM and 1 μM; HS^−^: 1 nM and 10 mM). Calculations involving assumed concentration minima and maxima were used to assess the energetic feasibility of hydrogenotrophic and methylotrophic methanogenesis reactions and AOM, and the conditions or locations where these reactions were most likely to take place. Activities of all aqueous species were calculated from measured concentrations multiplied by their activity coefficients. These were γ_HCO32−_ = 0.532 (Millero and Schreiber, 1982), γ_CH4_ = 1.24 (Millero, 2000), γ_SO42−_ = 0.104 (Millero and Schreiber, 1982), and γ_HS−_ = 0.685 (Clegg and Whitfield, 1991). The activity coefficients of H_2_ and acetate were set to those of methane and bicarbonate, respectively. We assumed an activity of 1.0 for methanol. Following convention, the activity of H^+^ was equal to its pH-value, and the activity of H_2_O was set to 1.0. Standard Gibbs energies (∆G_f_°), standard enthalpies (∆H_f_°), and standard molal volumes (∆V_f_°) of formation are shown in Supplementary Table S1.

### Multivariate statistics

All statistical analyses were performed in R.^5^ Richness (the observed number of ZOTUs), Pielou’s Evenness (a measure of how similar the abundances of different ZOTUs were; Heip et al., 1998), and Non-metric Multi-Dimensional Scaling (NMDS) based on Bray-Curtis distances of *mcr*A communities between samples were calculated using the ‘phyloseq’ package (McMurdie and Holmes, 2013). PERmutational Multivariate ANalysis Of VAriance (PERMANOVA, permutations = 999) and statistical tests (Welch’s t test and Wilcoxon test) were performed using the “vegan” and “stats” packages, respectively (McMurdie and Holmes, 2013). Correlations between abundances of *mcr*A groups and environmental variables were calculated and visualized using the “corrplot” package (Wei et al., 2017). All calculations were performed based on ZOTU-level phylogenetic assignments unless stated otherwise.

## Results

### Geochemical profiles related to the sedimentary methane cycle

**Figure 2:**
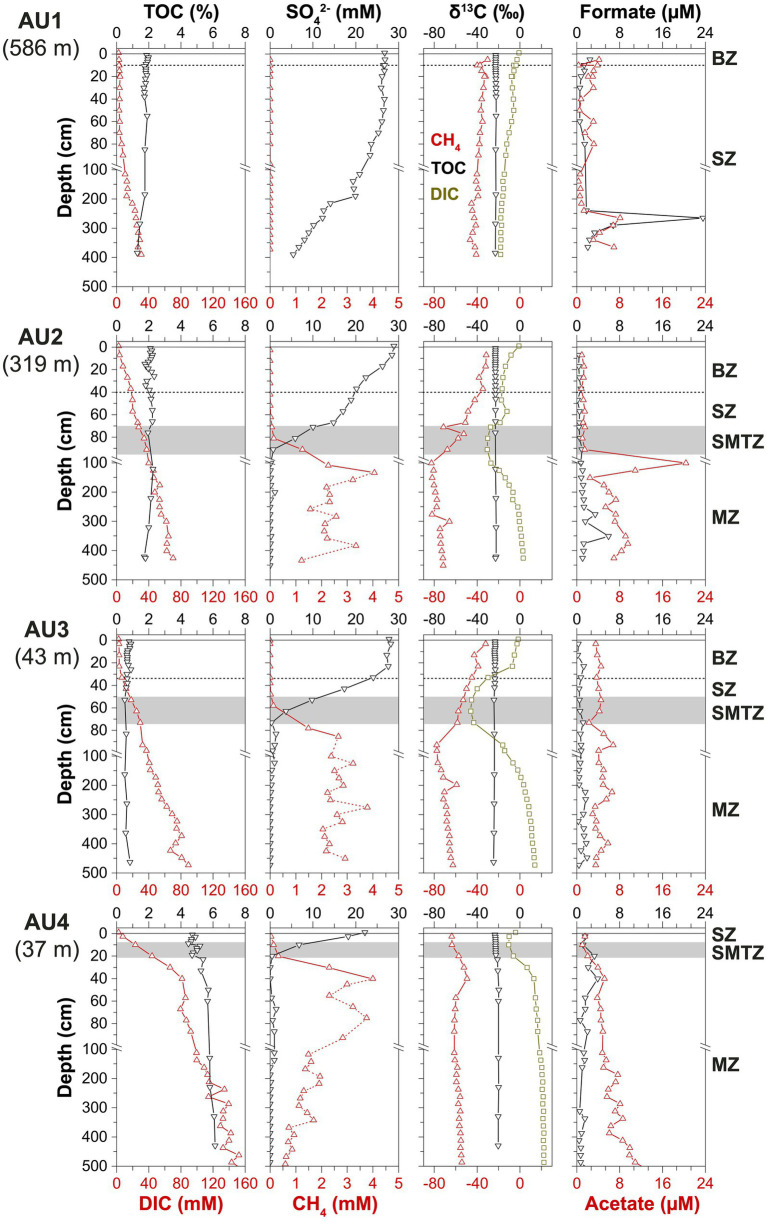
Depth profiles of TOC, DIC, SO_4_^2−^, CH_4_, δ^13^C-TOC, δ^13^C-DIC, δ^13^C-CH_4_, formate, and acetate at AU1-4. Depth intervals of the bioturbation zone (BZ), sulfatic zone (SZ), sulfate–methane-transition zone (SMTZ), and methanic zone (MZ) are indicated by horizontal dashed lines and gray bars, respectively. Due to out-gassing of CH_4_ from sediments with *in situ* CH_4_ concentrations >1 mM at atmospheric pressure during sampling, most measured CH_4_ concentrations below the SMTZ (symbols connected by the dotted lines) are likely to be significant underestimates and do not represent *in situ* concentrations.

The four stations show up to 10-fold differences in total organic carbon (TOC) contents, as well as an increase in microbial activity from deep to shallow stations (Figure 2). TOC contents (% sediment dry wt.) are highest at the sulfidic coastal station (AU4; 4.7–6%), lowest at the sandy shallow water station (AU3; 0.5–1%), and have intermediate values at the two deep stations (AU1: 1.3–1.9%; AU2; 1.7–2.1%). Corresponding DIC concentrations, used as a proxy for organic matter mineralization rates, indicate increases in DIC depth gradients from the deepest (AU1) to the shallowest station (AU4). This apparent increase in mineralization rates from deep to shallow water is also reflected in the depth of sulfate penetration, which decreases with water depth (AU1: >400 cm, AU2: 95 cm, AU3: 75 cm, AU4: 20 cm). Correspondingly, methane concentrations remain at background values (≤10 μM) throughout the sulfate-rich AU1 core but increase steeply to millimolar concentrations in the SMTZ at the three other stations. SO_4_^2−^ and DIC concentrations are nearly constant in the top 60, 10, and 25 cm of AU1, AU2, and AU3. This is explained by significant bioirrigation activity at these sites, and surface intervals with iron and manganese reduction-dominated microbial respiration at AU1 and AU2 (Kristensen et al., 2018; Deng et al., 2020).

The carbon stable isotopic compositions show clear trends across stations. The δ^13^C-TOC values throughout AU1 to AU3 and in the top 20 cm of AU4 are in the typical range of marine phytoplankton (−24 to −22‰; Fry and Sherr, 1989). Below 20 cm, the δ^13^C-TOC at AU4 increases slightly (−20‰ at 50 cm and below). The δ^13^C-CH_4_ at AU1 decreases from −30‰ at 5 cm to −41‰ at 390 cm. At AU2 and AU3, δ^13^C-CH_4_ values are also around −30‰ in surface sediments, but decrease throughout the SZ and SMTZ, reaching their lowest values right below the SMTZ (−80‰), before increasing slightly in deeper parts of the MZ (−70 to −60‰). In marked contrast, at AU4 δ^13^C-CH_4_ increases from −60‰ in the surface sediment to −50‰ at 40 cm and remains relatively constant below (−58 ± 2‰).

The δ^13^C-DIC profiles also show strong variations between sites. At all sites, δ^13^C-DIC values are near seawater values (0‰) in surface sediments. Yet, while at AU1 δ^13^C-DIC values show a gradual decrease with depth to −18‰ at 390 cm, all other stations have unimodal distributions, with the most negative δ^13^C-DIC values in the SMTZ (AU2: −30‰; AU3: −46‰; AU4: −10‰). In the uppermost section of the MZs, the δ^13^C-DIC increases steeply and leads to δ^13^C-DIC > 0‰ below a certain depth in the MZ (AU2: 3 m; AU3: 1.6 m; AU4: 0.2 m). δ^13^C-DIC at the bottoms of cores from these stations has values of +3‰ (AU2), +14‰ (AU3), and + 22‰ (AU4).

Concentrations of formate and acetate were generally in the low micromolar range (≤10 μM), with acetate concentrations exceeding those of formate in most samples. At AU1, both remain below 4 μM from 0 to 250 cm but show a strong subsurface peak at 265 cm (formate: 24 μM, acetate: 8 μM), below which concentrations drop again. At AU2, formate and acetate concentrations are <3 μM above the SMTZ. Below the SMTZ, formate concentrations increase down to 351 cm (formate: 6 μM; acetate: 9 μM) before decreasing again toward the core bottom (formate: 1 μM; acetate: 7 μM), and acetate shows an additional peak in the uppermost sample of the MZ (20 μM; 101 cm). By comparison, formate and acetate concentrations are more constant with depth at AU3 (formate: 0.9 ± 0.5 μM, acetate: 4 ± 1 μM). At AU4, both formate and acetate concentrations increase from 1.5 μM at 5 cm to 4–5 μM at 40 cm. Below this depth, formate concentrations gradually decrease to 0.7 μM, while acetate concentrations gradually increase to 11 μM.

### Depth-related trends in absolute and relative abundances of *mcr*A copies

**Figure 3:**
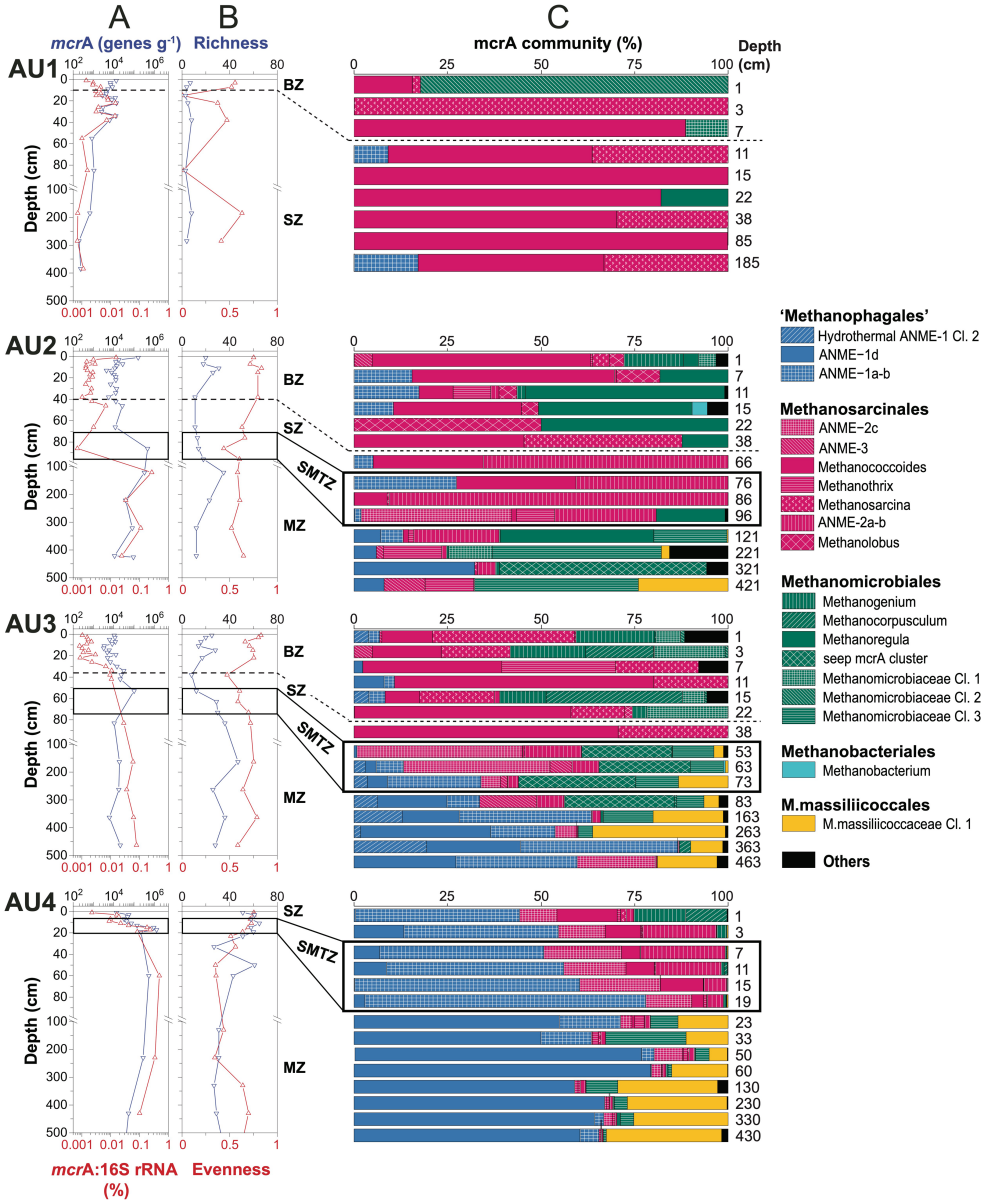
Depth profiles of methane-cycling archaeal communities at AU1-4. **(A)** *mcr*A gene copy numbers per gram wet sediment and ratios of *mcr*A to total 16S rRNA gene copy numbers; **(B)** richness and evenness based on *mcr*A ZOTUs clustered at 97% similarity level; **(C)** *mcr*A community composition at the genus-level. Samples above the horizontal dashed lines were located within the bioturbation zone. Black boxes indicate samples that were located within the sulfate–methane transition. BZ, bioturbation zone; SZ, sulfatic zone; SMTZ, sulfate–methane transition zone; MZ, methanic zone.

Copy numbers of *mcr*A are similar in surface sediments of all sites, independent of whether these are bioturbated and have an oxidized surface layer (AU1-3) or not (AU4). Yet, copy numbers increase from the oligotrophic AU1 to the eutrophic AU4 when deeper layers are compared. In addition, *mcr*A copies increase from the SMTZ into the underlying MZ at AU2-4, suggesting net population growth of methane-cycling archaeal populations after sediment burial (Figure 3A).

At AU1, *mcr*A abundances fluctuate around 10^4^ gene copies g^−1^ within the upper 40 cm and decrease to ~10^2^ gene copies g^−1^ below. At AU2, except for a high value at the sediment surface (~10^5^ genes g^−1^), *mcr*A copies are relatively stable (~10^4^ genes g^−1^) within the bioturbated upper 40 cm, below which they increase to 5 × 10^5^ copies g^−1^ at and right below the SMTZ, and then decrease gradually to ~10^4^ g^−1^ at the core bottom. At AU3, *mcr*A copies are ~10^4^ g^−1^ in the strongly bioturbated top 20 cm, increase to ~10^5^ g^−1^ at 53 cm and are relatively stable around 10^4^ g^−1^ below. At AU4, *mcr*A copies increase from ~10^4^ g^−1^ at the sediment surface to ~10^6^ g^−1^ around the SMTZ (20 cm) and are relatively constant below (~10^5^ g^−1^).

Abundances of methane-cycling archaea relative to total microbial abundances were estimated based on ratios of *mcr*A to total 16S rRNA gene copy numbers (bacterial+archaeal). These ratios suggest (local) increases in the relative abundances of methane-cycling archaea with sediment depth at all 4 locations. At AU1, which has no MZ, these increases are restricted to sediments near and right below the bottom of the BZ (1 cm: 0.001%; 22 cm: 0.015%), below which they decrease back to 0.001%. At the other three stations, relative abundances are in the same range as at AU1 in surface sediment (0.001%) and also increase at the bottom of the BZ (AU2, AU3). An additional increase occurs further down across the SMTZ into the uppermost MZ, where maximum values of 0.3% (AU2), 0.1% (AU3), and 0.5% (AU4) are reached. While relative abundances of methane-cycling archaea were highest at the core bottom of AU3, relative abundances at AU2 and AU4 decreased again toward the core bottom to values of 0.01% (AU2) and 0.1% (AU4).

### Zonation of major methane-cycling archaeal clades in relation to sites and vertical zones

*mcr*A richness calculated based on ZOTUs increases from AU1 to AU4 (Figure 3B). At AU1, ZOTU richness is low remaining <10 at all depths. The other locations have higher richness, and have local peaks of ≥30 ZOTUs in the BZ and/or upper MZ. Pielou’s Evenness, which was also based on ZOTUs, is on average lowest with high scatter at AU1, and is stable with slight depth-related decreases at the other stations. Overall, evenness is highest at AU3.

**Figure 4:**
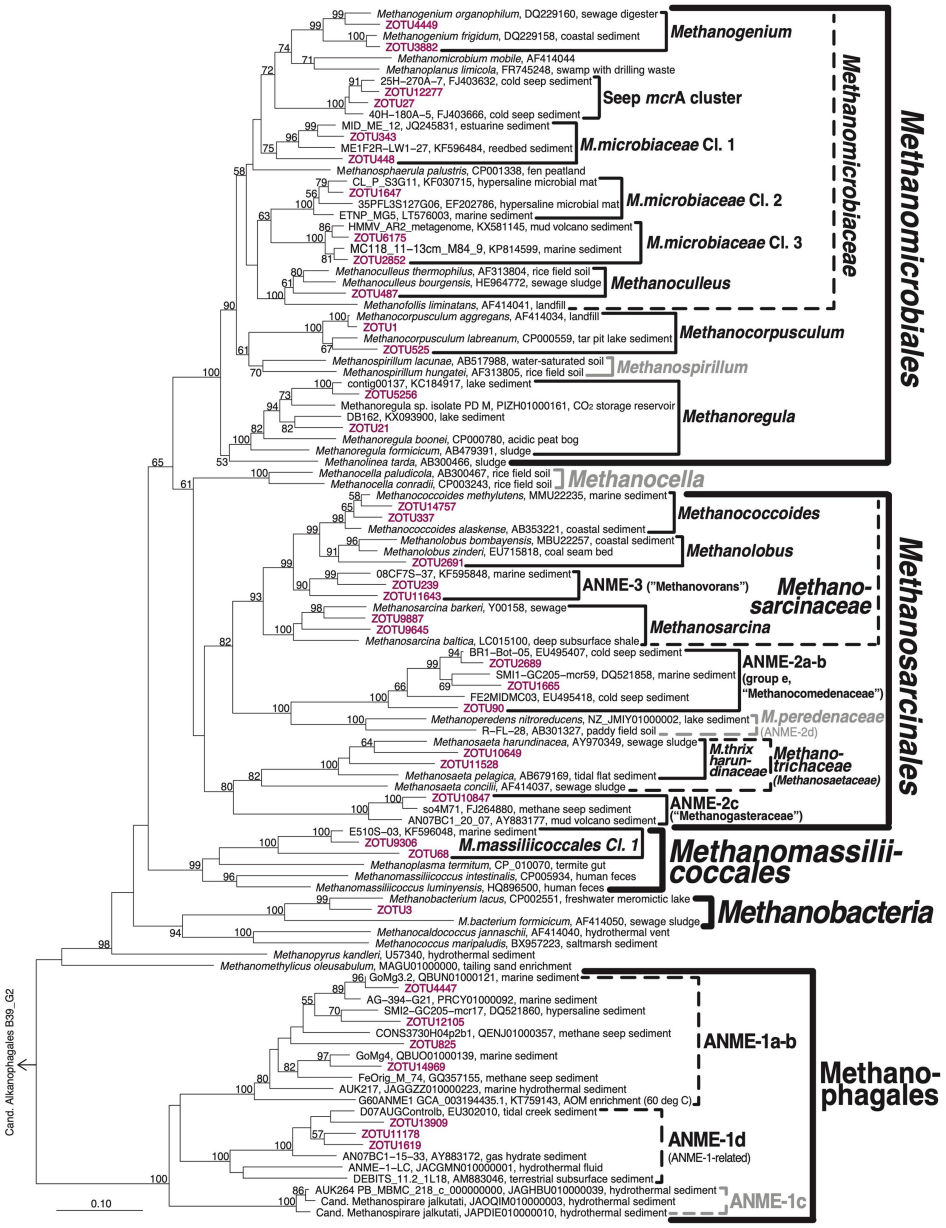
Phylogenetic tree of *mcr*A clades. Representative ZOTUs of environmentally important clades are shown in magenta. Latin names of proposed candidate taxa based on genomic analyses are placed in quotes. Clades that were not detected but included for illustration purposes are shown in gray. ‘Hydrothermal ANME-1 cluster’ (represented by ZOTU825) is treated as separate from ANME-1a-b in Figure 3 and in the text because of its clear phylogenetic separation from ANME-1a-b in more extensive, sequence-rich *mcr*A trees.

The community composition of methane-cycling archaea varies greatly across sites and in relation to the BZ, SZ, SMTZ, and MZ (Figure 3C; for taxonomic assignments see phylogenetic tree in Figure 4). Diverse genera of *Methanosarcinales* and *Methanomicrobiales* dominate throughout AU1 and AU2, and down to the SMTZ of AU3, while the Candidate order Methanophagales (ANME-1) dominates the lower part of the SMTZ and MZ of AU3 and AU4. Notably also, *Methanomassiliicoccales* account for major fractions (~10–35%) of *mcr*A reads throughout the MZs of AU3 and AU4 and at the bottom of AU2.

At the genus- and family-level we observe additional site- and geochemical zone-related trends (also see Figure 4 and next section). AU1 is dominated by a new cluster of *Methanomicrobiales* (*Methanomicrobiaceae* Cluster 2) at the sediment surface. Throughout the remaining core, sequences belonging to *Methanococcoides* and *Methanosarcina* (both *Methanosarcinaceae*) dominate, with minor contributions of ANME-1a-b, *Methanoregula* and *Methanomicrobiaceae* Cl. 1 in a few layers.

*Methanococcoides* and *Methanolobus* dominate the BZ and SZ of AU2 along with *Methanoregula* and *Methanogenium*. A major shift occurs in the SMTZ, where sequences of the family-level ANME-2a-b (also known as ‘group e’ or Candidatus Methanocomedenaceae), a sister clade of anaerobic methane-oxidizing *Methanoperedenaceae* (both *Methanosarcinales*), dominate, along with methanotrophic ANME-2c (Methanogasteraceae) (one sample only). A second shift occurs below in the MZ, toward a heterogeneous assemblage dominated by the new *Methanomicrobiaceae* Cluster 3, seep *mcr*A cluster (Lever and Teske, 2015), and *Methanoregula* (all *Methanomicrobiales*). Notably, *Methanothrix* (also known as *Methanosaeta*) account for significant percentages (~10–15%) in several layers, as do ANME-1d, a sister clade of ANME-1a-b [(Lever et al., 2023); for further information, see next section]. ANME-1a-b are also abundant at AU2, but mainly in and above the SMTZ.

*Methanococcoides*, *Methanosarcina*, *Methanogenium* and *Methanomicrobiaceae* Cl. 1 (also) dominate the BZ and upper SZ of AU3, along with *Methanocorpusculum*. As at AU2, there is a clear community shift in the lower SZ and SMTZ, where ANME-2a-b, ANME-2c, ANME-3 (“Candidatus Methanovorans”), seep *mcr*A cluster, and *Methanomicrobiaceae* Cl. 3 become dominant. In the lower part of the SMTZ the community shifts again, becoming dominated by ANME-1a-b, ANME-1d, and *Methanomassiliicoccales* in the MZ.

The vertical zonation at AU4, which has no bioturbation zone, is distinct from the other sites. ANME-1-a-b dominates the SZ and SMTZ and is replaced by ANME-1d in the upper part of the MZ. The SZ and SMTZ additionally have significant percentages of ANME-2a-b, ANME-2c, and *Methanococcoides*, while *Methanogenium* and *Methanocorpusculum* contributely to communities in surface sediment. In addition to ANME-1d, *Methanomassiliicoccales* and to a lesser degree *Methanomicrobiaceae* Cl. 3 and ANME-2c are relatively abundant in the MZ.

### *mcr*A phylogeny

A phylogenetic tree confirms the high diversity of methane-cycling archaeal taxa at the four sites (Figure 4). Most of this diversity is within the *Methanomicrobiales* and *Methanosarcinales*. While uncharacterized clusters dominate the former, the latter are dominated by metabolically well-characterized groups. Phylogenetic diversity is considerably lower within the ANME-1/Methanophagales, the *Methanomassiliicoccales* (class *Thermoplasmata*), and *Methanobacteria*.

In terms of phylogenetic diversity, the *Methanomicrobiales* are dominated by *Methanomicrobiaceae*, which comprise six of the eight detected *Methanomicrobiales* clusters. Within the *Methanomicrobiaceae*, the genera *Methanogenium* and *Methanoculleus* have cultured members, whereas the Seep *mcr*A cluster and newly proposed *Methanomicrobiaceae* Clusters 1–3 are only known from environmental samples, including methane seeps (Seep *mcr*A cluster) and a range of marine sedimentary habitats (*Methanomicrobiaceae* Cl. 1–3; Figure 4). Since all cultured members of *Methanomicrobiaceae* perform methanogenesis by CO_2_ reduction using H_2_ or formate as electron donors (Whitman et al., 2014), the four uncharacterized *Methanomicrobiaceae* likely also perform these reactions. The remaining groups consist of close relatives of *Methanocorpusculum aggregans* and a subcluster of *Methanoregula*. Cultured members of both groups reduce CO_2_ using H_2_ and/or formate as electron donors (Whitman et al., 2014).

The *Methanosarcinales* groups present consist of *Methanococcoides* and *Methanolobus*, which grow by disproportionation of methanol and methylamines (both genera), and several additional C1 compounds (certain *Methanolobus*; Oremland and Boone, 1994, Liang et al., 2022). The closely related ANME-3 group is considered to be methanotrophic (Bhattarai et al., 2019). *Methanosarcina* are substrate generalists, known to produce methane from H_2_/CO_2_, acetate, methanol, methyl sulfides, and methylamines, but not formate (Whitman et al., 2014). In addition, members of this group can grow by CO_2_ reduction via extracellular electron transfer (EET) from minerals or syntrophic partners (Rotaru et al., 2014; Gao and Lu, 2021). Outside of the *Methanosarcinaceae* the three additional groups of *Methanosarcinales* include ANME-2a-b (also known as group *e*), a sister clade of *Methanoperedenaceae*, members of which use nitrate-, iron(III)-, and manganese(IV) as electron acceptors for AOM (Haroon et al., 2013; Ettwig et al., 2016). Members of ANME-2a-b have been widely reported from methane seeps with AOM (e.g., Hallam et al., 2003; Lloyd et al., 2006). In addition, the known methanotrophic ANME-2c group (also known as ‘group c-d’; Knittel and Boetius, 2009) is present along with aceticlastic *Methanotrichaceae* (also known as *Methanosaetaceae*; Oren, 2014). Within the latter, all sequences fall into a genus-level cluster with the previously isolated *Methanothrix pelagica* and *Methanothrix harundinaceae*. Notably, *Methanothrix harundinacea*, similar to certain *Methanosarcina*, can also grow by CO_2_ reduction via DIET (Gao and Lu, 2021).

The community structure of *Methanophagales* consists of two major groups. ANME-1a-b is one of the most studied groups of methanotrophic archaea. Different from phylogenomic or 16S rRNA gene sequence analyses, ANME-1a cannot be reliably separated from ANME-1b based on *mcr*A sequence analyses (hence the name ANME-1a-b). In addition, we detect the recently named ANME-1d cluster (Lever et al., 2023). This cluster, which is also known as ANME-1-related group (Lever and Teske, 2015; Aromokeye et al., 2020), has been found in subseafloor sediments (Lever et al., 2023), serpentinitic hydrothermal vents (Kelley et al., 2005), deep coalbeds (Fry et al., 2009), and gas hydrate sediments (Kormas et al., 2008). ANME-1d is phylogenetically clearly distinct from ANME-1a-b (Figure 4), likely representing a separate family or even order (Lever and Teske, 2015).

The *Methanomassiliicoccales* detected here belong to a phylogenetic cluster that is distinct from the cultured genus *Methanomassiliicoccus* or the genome-sequenced candidate genera *Methanoplasma* or *Methanomethylophilus*. The closest relatives of this cluster based on *mcr*A phylogeny were detected in other (marine) sedimentary environments (e.g., KF596048, KF595850, AND KF595354 from Zhou et al., 2015). Based on pure culture and genomic evidence all three known genera of *Methanomassiliicoccales* are methanogens that reduce methanol or methylamines with H_2_ (Lang et al., 2015; Kröninger et al., 2017). Members of the CO_2_-reducing *Methanobacteriales*, which were only detected at significant abundances in one sample from the bioturbation zone of AU2, are represented by one phylotype (ZOTU3) that is most closely related to *Methanobacterium lacus*.

### Substrate use

**Figure 5:**
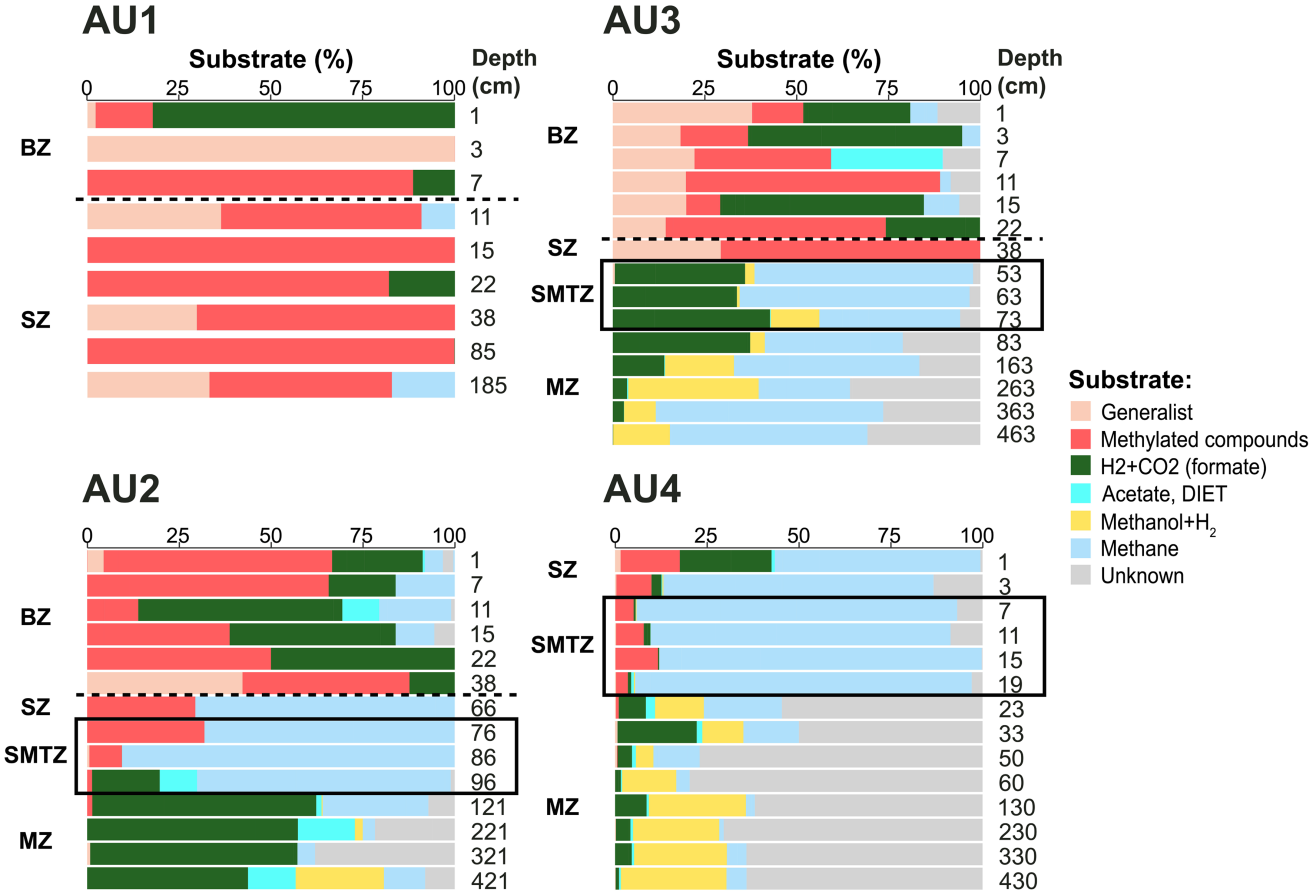
Depth profiles of major energy substrates at AU1-AU4, inferred from taxonomic identity, and related physiological knowledge, of major *mcr*A clades. In each sample, read percentages of all taxa (Figure 3) that use a specific substrate or substrate spectrum based on prior publications (Whitman et al., 2014; Lang et al., 2015; Gao and Lu, 2021; Chadwick et al., 2022, and references within) were summed into one substrate category. For instance, light blue reflects the combined read percentages of all known methanotrophs (ANME-1a-b, ANME-2a-b, ANME-2c, and ANME-3) from Figure 3. Similarly, “generalist” reflects the read percentages of *Methanosarcina*, the only known broad-substrate spectrum genus of methane-cycling archaea, from Figure 3. Samples above the horizontal dashed lines were located within the bioturbation zone. Black boxes indicate samples located within the sulfate–methane transition. BZ, bioturbation zone; SZ, sulfatic zone; SMTZ, sulfate–methane transition zone; MZ, methanic zone.

Known substrate uses of different methane-cycling archaeal groups provide insights into the distributions of methane-cycling pathways in the sediment (Figure 5). Taxa that perform methyl disproportionation were largely restricted to the BZ and SZ of AU1-AU3. By contrast, taxa that use methanol+H_2_, were largely absent from these layers, but increased to significant percentages in the MZ. Known CO_2_-reducing taxa showed rather patchy distributions, dominating AU2 except in the SMTZ, showing locally high percentages in all major zones of AU3, and being numerically abundant in surface and a few deeper samples of AU1 and AU4. Putative methanotrophs (ANME-1a-b, Hydrothermal ANME-1, ANME-2) accounted for high percentages in all SMTZs. In addition, these methanotrophs dominated the MZ of AU3 and SZ of AU4, and had locally significant contributions in the BZ and SZ of AU1-AU3. Remarkably, the energy substrates of a major, locally dominant, fraction of *mcr*A reads, belonging primarily to ANME-1d and uncultured *Methanomicrobiaceae*, are unknown.

### Zonation of *mcr*A community structure at the ZOTU-level

**Figure 6:**
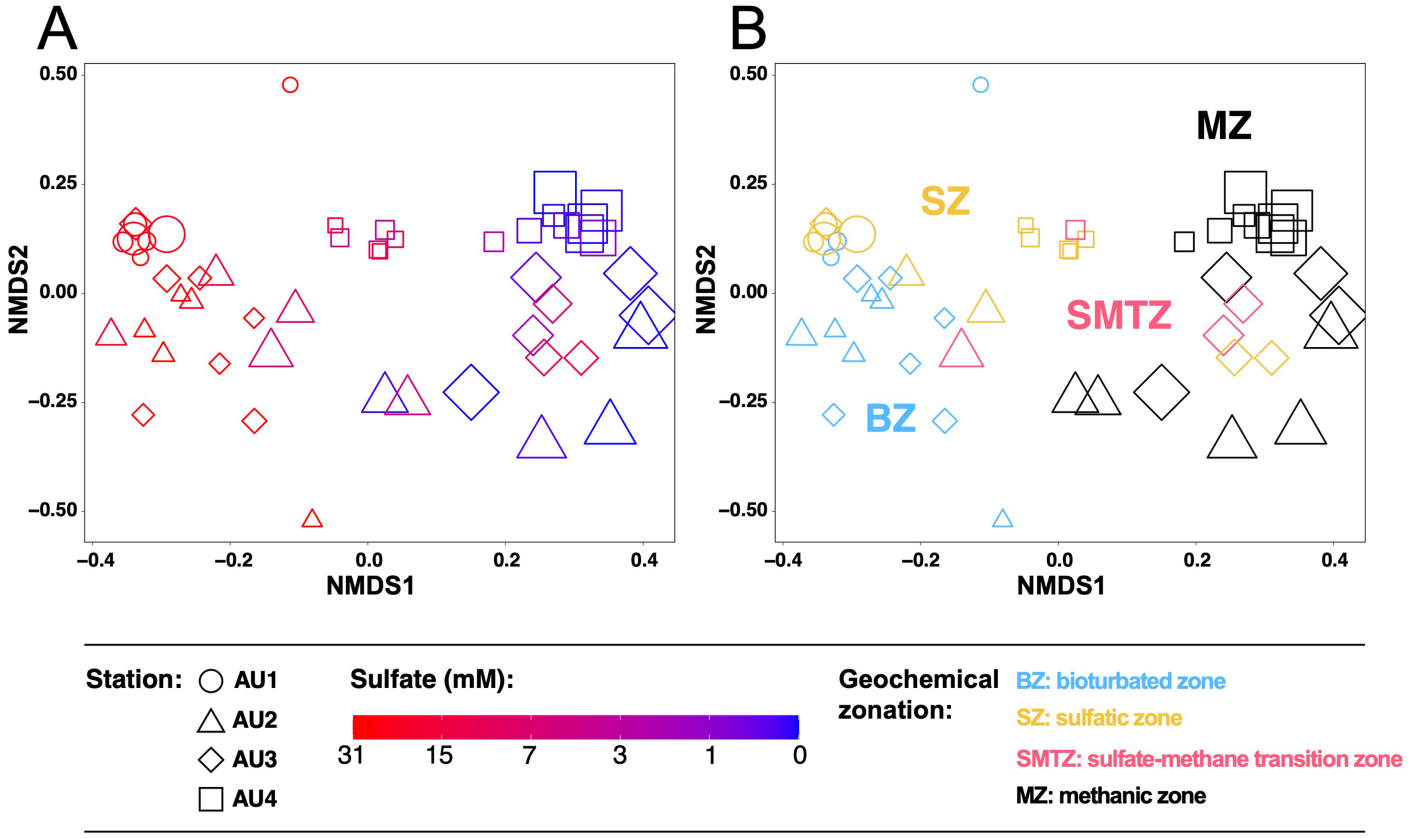
Non-Metric Multidimensional Scaling (NMDS) based on Bray-Curtis distances of *mcr*A communities at the 97% ZOTU-level in relation to **(A)** sulfate concentrations and **(B)** biogeochemical zone. Symbol sizes are proportional to sediment depth.

Analyses of methane-cycling archaeal distributions at the ZOTU-level show clear zonations also at the “species-level,” with sulfate concentrations as a likely key driver (NMDS1; Figure 6A). In addition, site-specific clustering can be observed (NMDS2). Community fingerprints overlap considerably between AU1, AU2, and AU3, but are more distinct in the sulfidic, more organic carbon-rich sediments of AU4. Examining ZOTU zonations in relation to biogeochemical zone (Figure 6B), communities in the BZ overlap strongly with those in the SZ but are mostly distinct from those in the MZ. By contrast, communities in the SMTZ, depending on location, variably cluster with samples from the BZ, SZ, or MZ.

## Discussion

By integrating geochemical, stable isotopic, and methane-cycling archaeal abundance and community data from coastal eutrophic to off-shore oligotrophic sites, we explore the drivers of methanogenesis and anaerobic oxidation of methane in continental margin sediments. Our analyses indicate that active methane-cycling is not restricted to sulfate–methane transition zones (SMTZs) and methanic zones (MZs), but also occurs in bioturbation zones (BZs) and sulfatic zones (SZs). Pervasive vertical and horizontal changes in methane-cycling communities and inferred metabolisms indicate a major role of depth- and site-specific environmental variables in driving the methane cycle in continental margin sediments.

### Distribution of methanogenic and methanotrophic activity

Downcore concentration profiles of methane, with low (micromolar) concentrations throughout the BZ and SZ, and increases to millimolar concentrations where sulfate is depleted in deeper layers, are consistent with the standard biogeochemical zonation (e.g., Jørgensen and Kasten, 2006; Figure 2). Accordingly, methanogenesis is suppressed by competing microbial reactions with higher energy yields, e.g., aerobic respiration, sulfate and metal reduction, in sediments where O_2_, sulfate and/or metal oxides are present. The distinct increase in methane concentrations in deeper, sulfate-depleted layers suggests that methanogenesis only becomes dominant once energetically superior respiration reactions are electron acceptor-limited. The steep drop in methane in the SMTZ is, moreover, consistent with sulfate-dependent AOM consuming upward-diffusing methane from the MZ in sediment layers where this methane overlaps with sulfate diffusing downward from overlying seawater.

While the concentration profiles of sulfate and methane indicate peak methane-cycling activity in the SMTZ and MZ, isotopic data suggest that active methane cycling, with *in situ* oxidation and possibly production, is also present in the BZ and SZ. At AU1 and AU2, δ^13^C-CH_4_ values increase from the MZ to the overlying SZ and BZ. Gradual oxidation of methane that has escaped oxidation in the SMTZ and is diffusing up into the SZ may drive this shift to higher δ^13^C-CH_4_. Herein the amount of isotopically “light” methane that is oxidized to DIC is too small to significantly lower the δ^13^C-DIC, given the much larger pool size of DIC from organic matter mineralization (Figure 2). In addition, “cryptic” methanogenesis, i.e., methane production without a clear imprint on methane concentrations due to simultaneous methane consumption by AOM, may occur throughout the SZ and BZ. The nearly parallel δ^13^C-CH_4_ and δ^13^C-DIC profiles throughout AU1, and in certain surface sedimentary intervals of AU2 and AU3, are consistent with low rates of CO_2_ reduction, wherein the δ^13^C-CH_4_ follows the δ^13^C-DIC with a fixed offset due to a constant isotopic fractionation factor during the conversion of CO_2_ (DIC) to methane. Similarly, low rates of other methanogenic reactions, e.g., acetate and methyl group disproportionation, cannot be ruled out based on the C-isotopic data. Fractionations produced by these reactions could be masked by other C-isotopic fractionations related to methane cycling.

Further down in the SMTZ, the dominant methane cycling reactions become more evident. In the SMTZ of AU2 and AU3, the slight increase in δ^13^C-CH_4_ and strong decrease in δ^13^C-DIC to values below those of δ^13^C-TOC indicate oxidation of isotopically light methane as a major source of DIC. Notably, AU4 shows a different trend. Despite the strong upward decrease in methane concentrations across the SMTZ, the δ^13^C-CH_4_ and δ^13^C-DIC both show parallel decreases toward the seafloor. We propose that, at AU4, significant rates of methanogenic CO_2_ reduction co-occur with AOM within the SMTZ. Despite the clear decrease in methane concentrations from the MZ up through the SMTZ, which suggest net oxidation of methane, the stronger negative isotopic fractionation associated with methanogenic CO_2_ reduction appears to dominate δ^13^C-CH_4_ values over the comparatively weaker isotopic fractionations associated with AOM. This interpretation matches past studies that indicate considerably higher C fractionations associated with methanogenic CO_2_ reduction (*α* = 1.045–1.082; reviewed in Conrad, 2005) than with AOM (*α* = 1.004–1.021; reviewed in Alperin and Hoehler, 2009). A similar co-occurrence of methane production and AOM in the SMTZ was previously proposed for organic-rich coastal sediments based on radiotracer incubations (Beulig et al., 2018) and C-isotopic analyses of methane-cycling microbial aggregates within SMTZs (Alperin and Hoehler, 2009). In addition, given the shallow sediment depth of the SMTZ at AU4 (~10 to 20 cm), it is possible that the strong decrease in methane concentration near the sediment surface is not solely caused by AOM, but additionally by methane diffusion or even ebullition into overlying water. Similar processes were recently proposed to explain “methane leakage” in eutrophic lakes (van Grinsven et al., 2022) and in organic-rich sediments of the eastern Baltic Sea (Hermans et al., 2024; Lapham et al., 2024). In the lake study, lower energy yields of methane oxidation compared to organotrophic reactions involving oxidation of amino acids, sugars, and certain VFAs were proposed to result in methanotrophs being outcompeted by organotrophs for shared electron acceptors in surface sediments of eutrophic lakes.

Measured δ^13^C-CH_4_ and δ^13^C-DIC profiles in the MZs of AU2 through AU4 are nearly parallel and increase with depth, consistent with CO_2_ reduction as the dominant methanogenic pathway. The offset between δ^13^C-CH_4_ and δ^13^C-DIC is remarkably constant between sites, mostly ranging from −68.2 to −80.5 per mil, which corresponds to an isotopic fractionation factor (α) of 1.075–1.085 for methanogenic CO_2_ reduction. These values are among the highest reported from marine sediments (Conrad, 2005). At all three sites, the rates of methanogenic CO_2_ reduction are high enough to drive δ^13^C-DIC into the positive range, to values that are up to +40 per mil higher than the δ^13^C-TOC (AU4).

### Net growth of methane-cycling archaea in response to vertical geochemical gradients

Copy numbers of *mcr*A are similar in surface sediments of all sites but are uniformly low in absolute abundances (10^2^–10^4^ copies g^−1^ sediment) and relative abundances, with *mcr*A:16S rRNA gene ratios of 10^−5^–10^−4^ (0.001–0.01%) (Figure 3A). Comparing deeper layers, there is, however, a clear increase in copy numbers from the oligotrophic AU1 to the eutrophic AU4. This trend matches the increase in sedimentary organic carbon content and reactivity from the deep Norway Trench site (AU1, 586 m) to the southern slope of the Norway Trench (AU2, 319 m) and shallow shelf sites, which include the sandy Kattegat site (AU3, 43 m) and muddy, sulfidic Lillebælt site (AU4, 37 m; Kristensen et al., 2018; Deng et al., 2020). Presumably, higher energy availability due to higher organic matter content and reactivity drives this increase in methane-cycling archaeal abundances in subsurface, sulfate-depleted layers from offshore to nearshore. Nevertheless, ratios of *mcr*A to total 16S rRNA gene copy numbers indicate that methane-cycling archaea account for only a small fraction (mostly <<1%) of the total microbial community, even in the SMTZ and MZ (Figure 3A). Thus, methane-cycling archaea appear to be part of a rare, albeit geochemically important, microbial biosphere in continental margin sediments. This observation is consistent with previously published data on these archaea from ocean drilling cores (Lever, 2013; Lever et al., 2023).

In addition to the trends in *mcr*A copy numbers across sites, there are clear vertical trends within sites. At AU1 through AU3, *mcr*A copy numbers increase at the bottom of the BZ, suggesting adverse effects of macrofaunal ventilation on methane-cycling archaeal community size. In addition, at AU2 through AU4, *mcr*A copy numbers increase by an order of magnitude from the lower SZ to the SMTZ and uppermost layer of the MZ. We interpret this as evidence of net population growth (i.e., cell division rates > mortality rates) in the past, when the low numbers of methane-cycling archaeal populations in SZs were buried from the SZ to the SMTZ and MZ, where geochemical regimes are more favorable due to high methane concentrations (methanotrophs) and reduced competition with sulfate reducers (methanogens) (Figure 3A). This interpretation is supported by data on methane-cycling archaea in subseafloor sediments of the Peru Trench. There, *mcr*A was below detection throughout the SZ, but became widely detectable in the SMTZ and MZ, suggesting net population growth of methane-cycling archaea thousands of years after sediment deposition, when geochemical conditions became more favorable (Lever et al., 2023).

### Methane-cycling archaeal communities of bioturbated and sulfate-rich sediments

**Figure 7:**
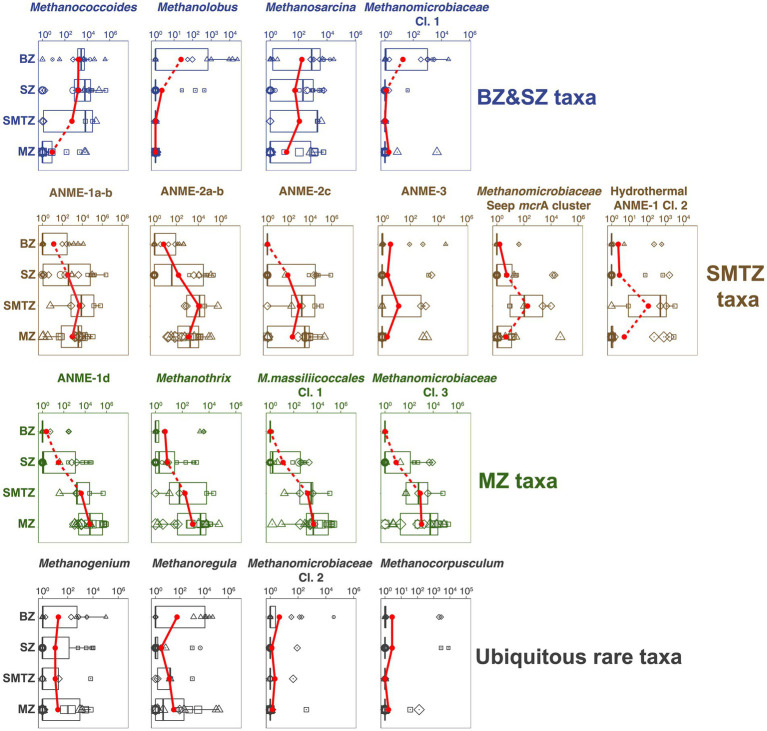
Vertical trends in absolute abundances of different methane-cycling archaeal groups in relation to geochemical zone. BZ, bioturbation zone; SZ, sulfatic zone; SMTZ, sulfate–methane transition zone; MZ, methanic zone. Sample-specific absolute abundances of *mcr*A genes (copies g^−1^ dry sediment) for each group were calculated by multiplying total *mcr*A copy numbers by the fraction of total *mcr*A reads contributed by that group. We observe four distinct trends: *BZ&SZ taxa, SMTZ taxa*, and *MZ taxa* refer to methane-cycling archaea with highest average abundances in the BZ and SZ, the SMTZ, and the MZ. A fourth category (*ubiquitous rare taxa*), with no clear depth trend and only low average abundances, is not further discussed. The average *mcr*A gene copy numbers within each geochemical zone are indicated by the red dots, whereas boxes indicate a 50% confidence interval. Dashed lines connecting adjacent points indicate significant differences of mean values (Wilcoxon test).

Phylogenetic analyses reveal diverse methane-cycling archaeal communities that vary with site location and in relation to vertical biogeochemical zones. Herein the biggest changes, both at the ZOTU-level and at higher phylogenetic levels, occur between sulfate-rich sediment (BZ + SZ), the SMTZ, and the MZ (Figures 3B,C, 6). These community changes are not only apparent at the relative abundance-level, but also when taxon-specific absolute abundances are compared across vertical biogeochemical zones (Figure 7).

A striking phylogenetic trend across the study sites is the shift from high percentages of *Methanosarcinales* in BZ, SZ, and SMTZ layers to much lower contributions in MZs. This indicates that elevated sulfate concentrations lead to higher contributions of *Methanosarcinales*. The reasons are offered by the vertical zonation of individual *Methanosarcinales* taxa. Specialized, methyl-disproportionating *Methanococcoides* and *Methanolobus* and generalistic *Methanosarcina* dominate both the BZ and SZ and decrease in relative and absolute abundances with depth (Figures 3C, 7). The fact that these taxa, which share the metabolic potential for methyl-disproportionation, dominate methanogenic communities in sulfate-rich sediments matches the notion that methylated compounds, such as methanol, methyl sulfides, and methylamines, are not utilized by most competing respiring organisms. This enables methylotrophic methanogens to thrive in sediments where sulfate or metal reduction dominate respiration (Maltby et al., 2015; Xiao et al., 2018; Zhuang et al., 2018).

In addition to *Methanosarcinales*, *Methanomicrobiales* account for a significant, in some places dominant, fraction of the methane-cycling archaeal community in sulfate-rich layers, with the highest percentages found in the BZ (Figure 3C). All cultivated members of this order, which includes the genera *Methanogenium*, *Methanocorpusculum*, and *Methanoregula* at AU2 through AU4, are obligate CO_2_-reducing methanogens (Garcia et al., 2006). Presumably, the novel genus-level lineages *Methanomicrobiaceae* Clusters 1 and 2, which were mostly found from AU1 to AU3, and which had their highest absolute abundances in BZs (Figure 7), are no exception. These significant percentages of *Methanomicrobiales*, and of *Methanosarcina* which also include facultative CO_2_ reducers, are in line with δ^13^C-CH_4_ and δ^13^C-DIC trends that suggest CO_2_ reduction in the BZ and SZ, but go against the notion that CO_2_-reducing methanogens are outcompeted by sulfate and metal reducers for H_2_ in these sediments (Lovley and Goodwin, 1988; Hoehler et al., 1998). We propose redox oscillations due to macrofaunal ventilation and episodic import of labile organic matter by macrofaunal reworking as potential reasons for the presence of CO_2_-reducing methanogens in BZs. The resulting fluctuations in redox conditions and electron donor supplies may prevent sulfate and metal reducers from drawing H_2_ concentrations down to steady-state levels that are too low to energetically support methanogenic CO_2_ reduction. Alternatively, CO_2_ reducers may not rely on H_2_, but instead use electrons from DIET, supplied by syntrophic partner organisms, to reduce CO_2_. CO_2_ reduction via DIET has been shown in laboratory studies with *Methanosarcina* (Holmes et al., 2018) and was recently proposed to dominate CO_2_ reduction by Methanomicrobiales in lakes (Meier et al., 2024).

Despite the upward decrease in methane concentrations from the SMTZ to the seafloor (Figure 2) and isotopic and genetic evidence for an active methane cycle in the BZ and/or SZ, only small subpopulations of putatively methanotrophic archaea were detected (0 to 20% of *mcr*A reads) at AU1-AU3 (mainly ANME-1a-b; Figure 3C). Potential reasons include the low net energy gains from sulfate-dependent AOM, which in many cases involves energy partitioning between methanotrophic archaea and sulfate-reducing partner organisms and may only support very small populations of methanotrophs. In addition, it is possible that in the BZs of AU1 through AU3, which receive episodic input of O_2_ and nitrate due to macrofaunal ventilation (Deng et al., 2020), physiologically more resilient methane-oxidizing bacteria (MOB) outcompete ANMEs for methane. This possibility, which has also been proposed for lake sediments (van Grinsven et al., 2022), is underscored by clear outnumbering of ANMEs by MOBs based on functional gene copy numbers. Copy numbers of the alpha subunit of partial methane monooxygenase (*pmo*A), a key gene of aerobic methane oxidation, are in the range of 10^5^ to 10^7^ copies g^−1^ sediment in the BZs (Supplementary Data File 1), and thus orders of magnitude higher than *mcr*A copy numbers (Figure 7). An exception is the non-bioturbated, mostly sulfidic surface sediment of AU4. Here ANME-1a-b and ANME-2 collectively account for >50% of *mcr*A reads in the SZ. We propose that due to the high methane flux and shallow depth of the SMTZ (5–20 cm) at AU4, significant amounts of methane escape oxidation in the SMTZ and are (partially), consumed by ANMEs in the overlying, only 5 cm thick SZ.

### Methane-cycling archaeal communities of sulfate–methane transition zones

We detect all known ANMEs, except *Methanoperedenaceae*, in the sediments studied. While there are taxonomic overlaps, each of the three SMTZs is dominated by a different ANME group. AU2 is dominated by ANME-2a-b (Candidatus Methanocomedenaceae), AU3 by ANME-2c (Candidatus Methanogasteraceae; both *Methanosarcinales*), and AU4 by the ANME-1a-b family (Candidatus Methanophagaceae, Candidatus Methanophagales). Moreover, while ≥80% of *mcr*A read percentages at AU2 and AU4 belong to ANMEs, only about half of the reads in the SMTZ of AU3 belong to ANMEs. The other half consist largely of uncultured *Methanomicrobiales* (seep *mcr*A cluster, *Methanomicrobiaceae* Cl. 3).

The dominance of anaerobic methanotrophs in SMTZs is expected, and the preference of these groups for SMTZs is supported by the fact that absolute abundances of all ANME groups except ANME-1d (discussed in next section) were highest in SMTZ (Figure 7). Yet, the reasons for the differences in dominant groups between locations are unclear. The fact that ANME-2a-b, ANME-2c, and ANME-1a-b co-occur in significant percentages in each location (Figure 3C) argues against dispersal limitation or competitive exclusion over a limiting resource. Instead, niche differences that reflect location-specific environmental variables may result in the dominance of different groups. While previous studies have suggested that ANME-2 thrive at higher sulfate concentrations than ANME-1 (Knittel and Boetius, 2009; Yanagawa et al., 2011), we observe significant contributions of ANME-1a-b at high sulfate concentrations in the SZ of AU4 and BZ of AU2 (Figure 2). Given that iron and manganese reduction are dominant respiratory reactions at AU2 and AU3 (Kristensen et al., 2018), AOM coupled to metal reduction may also take place and locally select for ANME-2a-b and ANME-2c taxa. This would be consistent with past research suggesting at least the involvement of ANME-2a in iron-dependent AOM (Aromokeye et al., 2020; Slobodkin et al., 2023). While ANME-1 and ANME-2 strongly overlap in distributions within SMTZs, and even in SZs, only ANME-1a-b and its sister clades ANME-1d and Hydrothermal ANME-1 Cluster 2 were detected at high abundances in sulfate-depleted MZs (AU3 and AU4). This matches the notions that ANME-1 are less dependent than ANME-2 on sulfate as an electron acceptor (Yanagawa et al., 2011) and include facultative methanogens (Lloyd et al., 2010; Beulig et al., 2018; Lever et al., 2023).

Even though the sulfate–methane concentration gradients indicate that almost all (AU2 and AU3) or at least a significant portion (AU4) of the methane that is produced in the MZs is consumed in the overlying SMTZs, there are several inconsistencies between our isotopic and genetic data. For instance, while AU3 has C isotopic profiles in the SMTZ that indicate AOM as by far the dominant methane-cycling process, we also detect high abundances of putatively CO_2_-reducing *Methanomicrobiales*. One possible explanation is that cell-specific activities of ANMEs are far higher than those of CO_2_-reducing *Methanomicrobiales*, but that only small populations of ANMEs are supported due to the low energy yields of AOM. Alternatively, Seep *mcr*A cluster, which is often detected in hydrocarbon and methane seeps with AOM (Joye et al., 2010; Lazar et al., 2012; Lever and Teske, 2015), could engage in AOM. Lastly, *Methanomicrobiales* within the SMTZ might be dormant. While the first explanation is plausible, and the second difficult to rule out but at odds with known physiologies of *Methanomicrobiales*, the notion of dormancy contradicts the average absolute abundance of Seep *mcr*A cluster, which is highest within SMTZs, suggesting growth stimulation within sulfate–methane transitions (Figure 7).

Remarkably, the isotopic data at AU4, with parallel gradients in δ^13^C-CH_4_ and δ^13^C-DIC across the SMTZ, indicate that CO_2_ reduction occurs at significant rates in parallel to AOM at this site, even overriding the isotopic imprint of AOM. This CO_2_ reduction could be performed, at least in part, by ANME-1a-b, consistent with previous radiotracer-based evidence for CO_2_ reduction by this group in SMTZs (Beulig et al., 2019). In addition, or alternatively, the less studied ANME-1d group (also known as ANME-1-related group; Lever and Teske, 2015), which is also abundant in the SMTZ of AU4, could be involved in CO_2_ reduction. This would match the generally deeper distribution of this group compared to ANME-1a-b (Figure 3C), and the fact that ANME-1d dominate methanogenic subsurface sediments in other locations (Aromokeye et al., 2020; Lever et al., 2023).

### Methane-cycling archaeal communities of methanic zones

While δ^13^C-CH_4_ and δ^13^C-DIC profiles suggest CO_2_ reduction as the dominant methanogenic pathway in the MZs of AU2 through AU4, taxonomic compositions paint a confusing picture (Figure 3C). Known CO_2_-reducing methanogens (*Methanoregula*) dominate in only a single sample from AU2, while all other samples are dominated by uncultured *Methanomicrobiales*, putative methane oxidizers of the ANME-1 group (ANME-1a-b, ANME-1d, Hydrothermal ANME-1 Cl. 2) and a new family-level *Methanomassiliicoccales* cluster. When absolute abundances are considered, then at least ANME-1d, *Methanomassiliicoccales*, *Methanomicrobiaceae* Cl. 3, and the less abundant aceticlastic genus *Methanothrix* are likely methane producers (Figure 7). *mcr*A copy numbers of these groups increase ~100 to 10,000-fold from the BZ and SZ to more methane-rich layers of the SMTZ and MZ and are highly correlated with methane concentrations (Supplementary Figure S1; all with Spearman’s Rho>0.6, *p* < 0.001).

The fact that MZs are dominated by uncultured taxa highlights the need for more cultivation research on marine methanogens. Even within the *Methanomicrobiales*, which include 24 published isolated species, only 5 isolates (*Methanogenium cariaci*, *Methanogenium marisnigri*, *Methanogenium organophilum*, *Methanolacinia paynteri*, *Methanoculleus thermophilicum*) are from marine environments – with all being CO_2_ reducers of the family *Methanomicrobiaceae* (Garcia et al., 2006). Nevertheless, given that the dominant *Methanomicrobiales* taxon in the MZ, *Methanomicrobiaceae* Cl. 3, also falls within the *Methanomicrobiaceae*, a CO_2_ reducing methanogenic metabolism seems likely.

The uncertainty increases when the dominant groups of ANME-1 are examined. While, for ANME-1a-b, the *mcr*A copy number peak in the SMTZ is consistent with methanotrophy (Figure 7), the high copy numbers and read contributions of this group in the MZ support the notion that this group includes facultative CO_2_-reducing methanogens (Beulig et al., 2019; Lever et al., 2023). A similar case could be made for the Hydrothermal ANME-1 Cl. 2. This group, which was first classified in hydrothermal sediment of Guaymas Basin (Lever and Teske, 2015), despite also having an *mcr*A copy number peak in the SMTZ, shows an increase in read contributions from the SMTZ to the MZ of AU3. The conditions under which either group might switch to a methanogenic lifestyle are unclear and may not always be strictly controlled by biogeochemical zone, e.g., radiotracer experiments have indicated ‘cryptic’ methanogenesis by CO_2_ reduction by ANME-1a-b in an SMTZ with net methane oxidation (Beulig et al., 2019).

While the distributions of ANME-1a-b and Hydrothermal ANME-1 Cl. 2 support the idea of these groups being facultative methanogens, the ANME-1d group shows distributions that indicate a primarily, if not solely, methanogenic lifestyle. This group not only increases in read percentages within the MZs of AU2-AU4 and dominates *mcr*A reads in the MZ of AU4 (Figure 3C). Its *mcr*A copy numbers furthermore increase 10^3^-fold from the BZ to the SMTZ and by an additional factor of ~10 in the MZ (Figure 7). While any inferences regarding methanogenic pathway deserve caution in the absence of cultivation or genomic data, C isotopic signatures indicative of CO_2_ reduction as the dominant methanogenic pathway at AU4, where ANME-1d account for 60% of *mcr*A reads, are in line with this group being a methanogenic CO_2_ reducer at the sites studied. Our interpretation matches results from deep subseafloor sediments in which ANME-1d dominated methane-cycling archaeal communities in layers that were > 100 m below the SMTZ and had been buried below the depth of sulfate-depletion for at least 400,000 years (Lever and Teske, 2015). Notably, CO2 reduction was inferred to also be the dominant methanogenic pathway in these deep subseafloor sediments

Another striking observation is the strong *mcr*A copy number increase of an unclassified *Methanomassiliicoccales* cluster in MZs. This group was below detection in the majority of BZ samples, increased ~1,000-fold from the SZ to the SMTZ, and again 10-fold from the SMTZ to the MZ from the SMTZ into the MZ (Figure 7). While this novel group remains physiologically uncharacterized, phylogenetic clustering within the *Methanomassiliicoccales* suggests methanogenic methyl group reduction with H_2_ as a likely metabolism (Lang et al., 2015; Kröninger et al., 2017). Remarkably, the increase in absolute abundances correlates negatively with that of methyl-disproportionating taxa (*Methanococcoides, Methanolobus, Methanosarcina*; all *p* < 0.05). This suggests a switch from methyl disproportionation as the prime methylotrophic methanogenic pathway in sulfate-rich surface sediments to methyl-reduction in deeper, sulfate-depleted layers (also see next section). With *mcr*A copy numbers in the MZ that are only second to ANME-1d, and with *mcr*A read percentages exceeding 20% in many of the deeper methanogenic layers, we propose that methyl group reduction is an important, widely overlooked methanogenic pathway in deep methanogenic marine sediments.

While the overall dominance of CO_2_ reduction over other methanogenic reactions is in line with isotopic data from a wide range of marine sediments (Whiticar et al., 1986; Whiticar, 1999), the generally low contribution of aceticlastic methanogens of the family *Methanotrichaceae* (*Methanosaetaceae*) is surprising given that acetate can be expected to be a major end product of microbial fermentation also in methanogenic sediments (Conrad, 1999, 2020). However, low contributions of acetate to methane production were previously reported for other marine MZs (Heuer et al., 2009). Moreover, past research has revealed that even in methanogenic marine sediments with high rates of acetate production, methanogens are not the dominant acetate-utilizing microorganisms. Instead, syntrophic acetate-oxidizing microorganisms convert acetate to an end product (e.g., H_2_) that is then used by CO_2_-reducing methanogens (Beulig et al., 2018). Notably, we observe highly significant absolute and relative abundance correlations between ANME-1d, *Methanomassiliicoccales*, and *Methanomicrobiaceae* Cl. 3 with Atribacteria (Atribacterota; Supplementary Figure S2, in all cases, Rho >0.6, *p* < 0.001). Members of this bacterial phylum, which dominates MZs of AU3 and AU4 and was also detected in significant percentages (to 10%) in the MZ of AU2 (Deng et al., 2020), have been linked to syntrophic acetate oxidation with H_2_ and CO_2_ as end products (Webster et al., 2011; Gies et al., 2014; Jiao et al., 2024). It is thus possible that syntrophic associations between acetate-oxidizing Atribacteria and CO_2_-reducing (e.g., ANME-1d, Methanomicrobiaceae Cl. 3) and methanol-reducing methanogens (*Methanomassiliicoccales*) play a key role in driving methane production in MZs of the Skagerrak-Kattegat region, and perhaps elsewhere.

### Drivers of methanogenic pathway distributions

The observed taxa distributions suggest environmental variations in the distributions of methanogenesis and anaerobic oxidation of methane (Figures 3, 4) that are not always visible within measured chemical concentration profiles and isotopic compositions (Figure 2). To explore potential drivers behind this cryptic cycling of methane, we next take a thermodynamic approach with a focus on *in situ* energy yields of various reactions.

**Figure 8:**
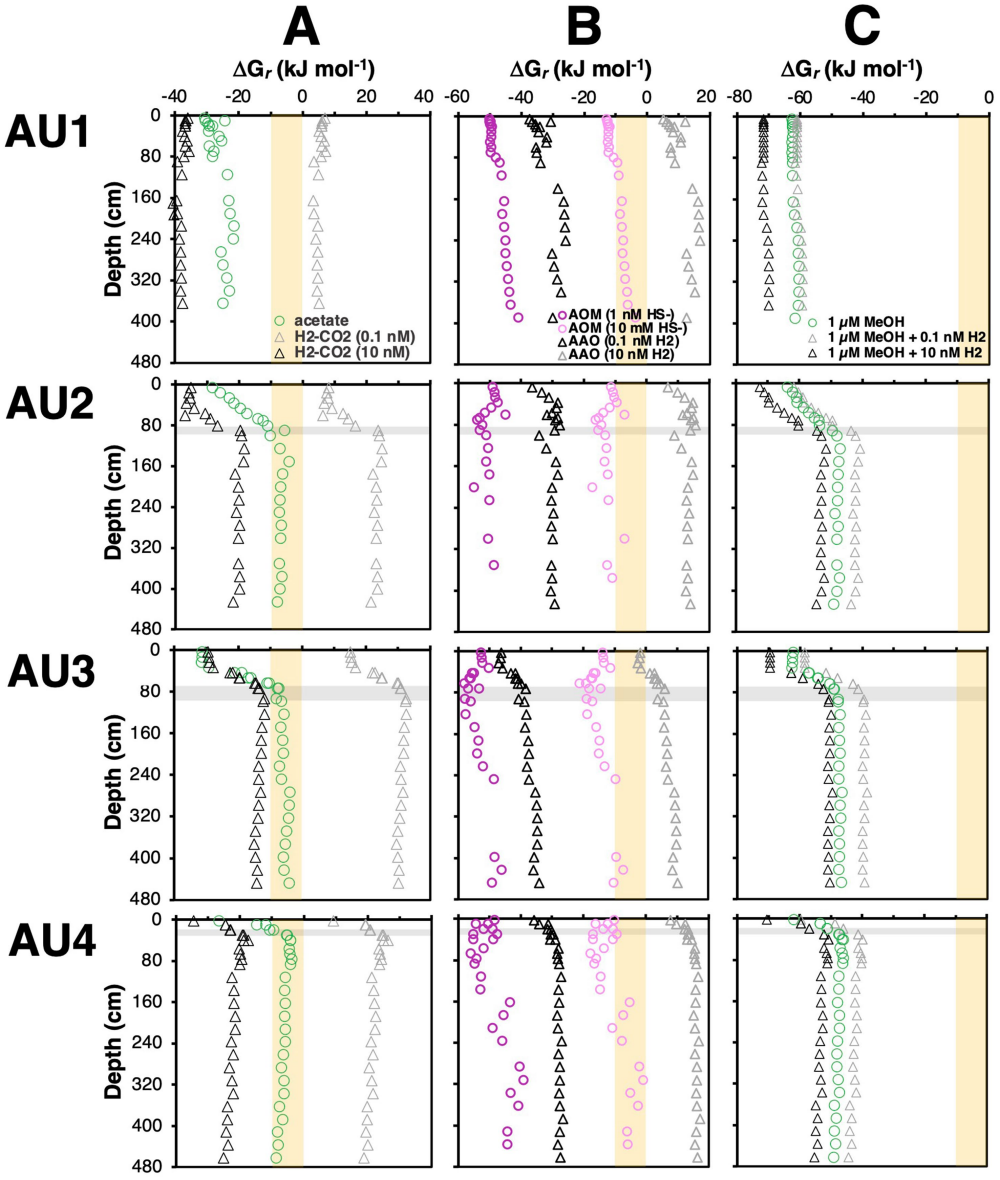
Calculated *in situ* Gibbs energies of **(A)** methanogenesis reactions involving hydrogenotrophic CO_2_ reduction (4 H_2_ + HCO_3_^−^ + H^+^ ➔ CH_4_ + 3 H_2_O) and aceticlastic methanogenesis (CH_3_COO^−^ + H_2_O ➔ CH_4_ + HCO_3_^−^), **(B)** sulfate-dependent AOM (AOM) (CH_4_ + SO_4_^2−^ ➔ HCO_3_- + HS^−^ + H_2_O) and anaerobic acetate oxidation (AAO) (CH_3_COO^−^ + 4 H_2_O ➔ 4 H_2_ + 2 HCO_3_^−^ + H^+^), and **(C)** methylotrophic methanogenesis via methanol disproportionation (4 CH_3_OH ➔ 3 CH_4_ + HCO_3_^−^ + H_2_O + H^+^) and methanol reduction (CH_3_OH + H_2_ ➔ CH_4_ + H_2_O). For reactions involving H_2_, we tested two scenarios ([H_2_] = 0.1 nM; [H_2_] = 10 nM). Gibbs energies for reactions involving methanol are per mol of methanol. Shaded beige areas indicate Gibbs energies that are exergonic but more positive (less energy-yielding) than the assumed biological energy quantum (ΔG*_r_* = −10 kJ mol^−1^ of reaction).

Given that H_2_ concentrations were not determined as part of this project, we test two H_2_ concentration scenarios. These H_2_ concentrations are in the range of the lowest ones found in anoxic sediments (0.1 nM) and in the typical range of methanogenic sediment (10 nM) (Lovley and Goodwin, 1988; Hoehler et al., 1998). We calculate that CO_2_ reduction is never exergonic at H_2_ concentrations of 0.1 nM, but always exergonic at concentrations of 10 nM, with Gibbs energies in the range of −40 to −12 kJ mol^−1^ (Figure 8A). Herein the most negative (exergonic) values were calculated for BZs and SZ, while the least negative (lowest energy yielding) values (−20 to −12 kJ mol^−1^ reaction) were calculated for MZs. These Gibbs energy values are close to the minimum biological energy quantum (BEQ) (−10 kJ mol^−1^; Hoehler et al., 2001) in a range previously determined for methanogens in methanogenic samples (Hoehler, 2004). The scenario is different in sulfate-rich sediments where, due to much lower concentrations of the end product methane, Gibbs energies are more negative. We estimate that H_2_ concentrations around 1 nM, i.e., halfway between Gibbs energies for [H_2_] = 0.1 nM and [H_2_] = 10 nM in Figure 8A, would be required to support methanogenic CO_2_ reduction via H_2_ in these sediments. Notably also, the Gibbs energies of CO_2_ reduction are in the same range in SMTZs as in MZs, suggesting that at 10 nM H_2_ methanogenic CO_2_ reduction is thermodynamically favorable in SMTZs. This could explain the isotopic evidence for CO_2_ reduction in the SMTZ of AU4 (Figure 2) and suggests that CO_2_ reduction by ANME-1a-b in SMTZs deserves consideration within the SMTZs of AU2 and AU3, despite sulfate-dependent AOM also being exergonic in these layers (Figure 8B). If CO_2_ reduction with H_2_ is indeed taking place in the SMTZs of these sites, then this makes the reversal of this reaction during AOM unlikely, and the H_2_-independent oxidation of methane via DIET a more likely scenario for AOM. The latter scenario is supported by genomic analyses of all three ANME groups, which lack genes for hydrogenases that would expectedly be needed for H_2_ production, but instead have the genomic potential for DIET (Chadwick et al., 2022).

**Figure 9:**
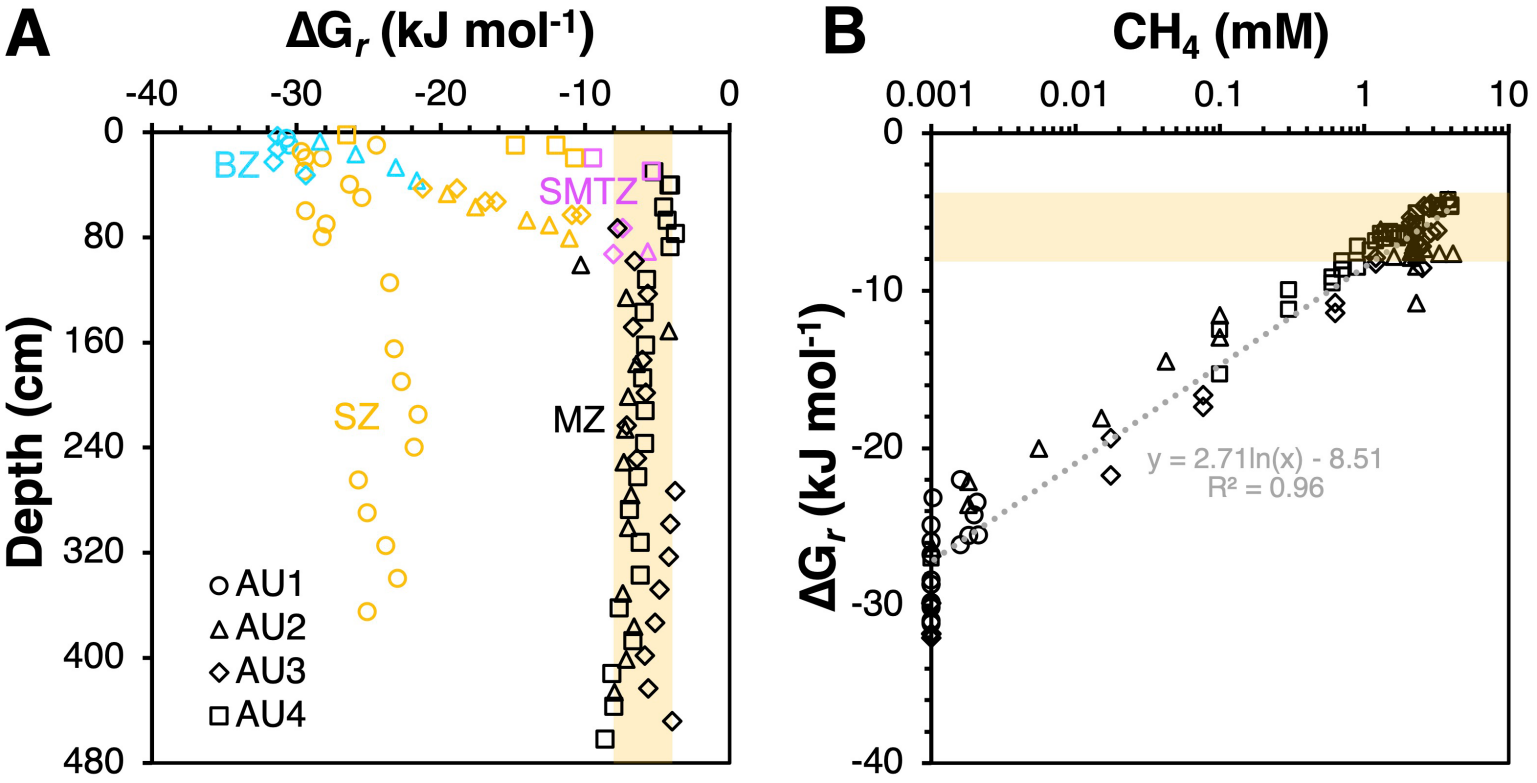
Observed relationship between Gibbs energies of aceticlastic methanogenesis versus **(A)** biogeochemical zone, and **(B)** measured methane concentrations. In **(A)** the color code is cyan for bioturbation zone (BZ), orange for sulfatic zone (SZ), magenta for sulfate–methane transition zone (SMTZ), and black for methanic zone (MZ). Note: measured methane concentrations in the MZ are likely to be underestimates due to supersaturation and resulting outgassing of methane during sampling at atmospheric pressure. Thus, actual Gibbs energies of aceticlastic methanogenesis in the MZ are likely to be less negative (less exergonic). Shaded beige areas indicate Gibbs energies in the range of −8 to −4 kJ mol^−1^, which included the vast majority of values.

We observe similar overall trends, i.e., higher free energy yields in BZs and SZs, and lower free energy yields in SMTZs and MZs, for aceticlastic methanogenesis. Acetate is often considered the most important energy substrate of sulfate-reducing bacteria in marine sediments (e.g., Parkes et al., 1989), and it is generally believed that sulfate reducers outcompete methanogens for acetate in the presence of high sulfate concentrations (King, 1984; Shaw et al., 1984; though also see Sela-Adler et al., 2017). Yet, calculated Gibbs energies of −30 to −10 kJ mol^−1^ reaction in SZs suggest that even in sulfate-rich sediments, aceticlastic methanogenesis is energetically feasible, with – perhaps surprisingly – higher free energy gains than in underlying MZs (Figures 8A, 9A), where buildup of methane results in less negative Gibbs energy values (Figure 9B). While the measured, micromolar acetate concentrations are too low for *Methanosarcina* based on previous physiological studies, due to the low-affinity acetate uptake system of the latter (Jetten et al., 1991; Berger et al., 2012), *Methanothrix*, which are known to thrive at acetate concentrations in the low micromolar range, occurred mainly in the SMTZs and MZs (Figure 7).

So why is aceticlastic methanogenesis not more strongly represented in the BZs and SZs given that this reaction is thermodynamically favorable? A potential explanation is the even higher energy yield (more negative Gibbs energy) associated with acetate oxidation coupled to sulfate reduction. The resultingly higher thermodynamic drive may benefit sulfate reducers via faster reaction rates. This, however, does not explain why acetate concentrations are not drawn down to lower concentrations, where Gibbs energies are closer to BEQ values of sulfate reduction or methanogenesis. Other factors, such as the energetic cost of acetate uptake could play a role. Unlike gases or its conjugate acid (acetic acid) – acetate anions cannot freely diffuse across the microbial cell membrane but require energetically costly active transport inside the cell (Casal et al., 2008). To be a viable energy source to sulfate reducers or methanogens, acetate catabolic reactions would need to provide sufficient energy not only to cover energetic costs, but also to produce a net energy gain. Expressed in protons (H^+^), average Gibbs energies may need to be sufficiently high to translocate two H^+^ per acetate anion, one for acetate uptake and another to contribute to ATP synthesis. The same reasoning could apply to sodium cations (Na^+^), assuming the use of a sodium motive force to conserve energy.

**Figure 10:**
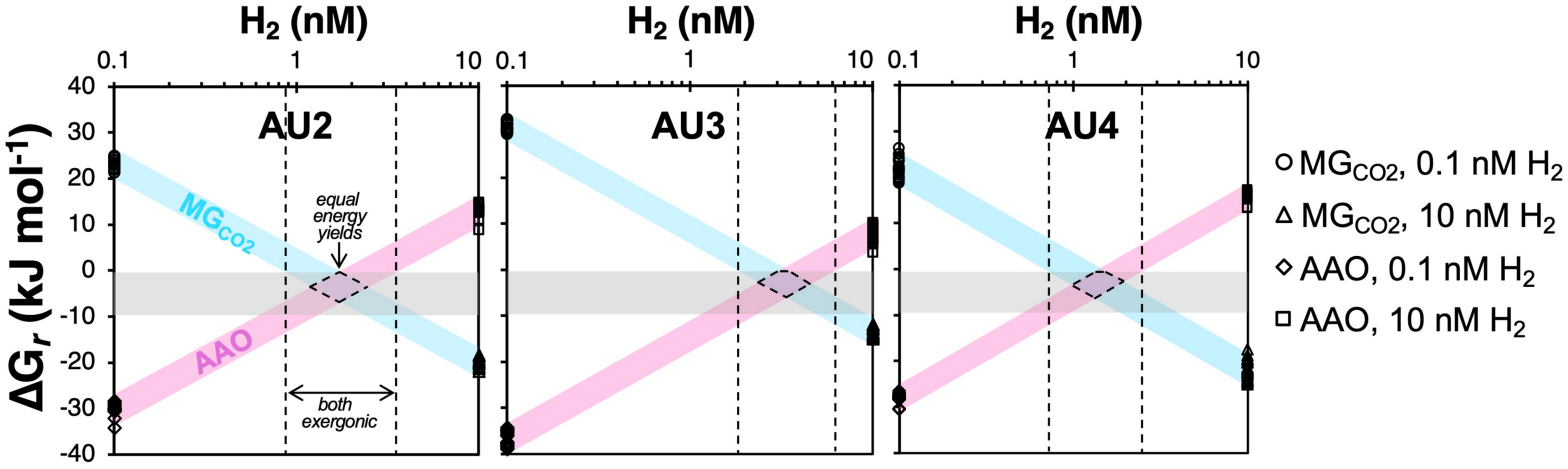
Calculated Gibbs energies of methanogenic CO_2_ reduction with H_2_ (MG_CO2;_ 4 H_2_ + HCO_3_^−^ + H^+^ ➔ CH_4_ + 3 H_2_O) and anaerobic acetate oxidation (AAO; CH_3_COO^−^ + 4 H_2_O ➔ 4 H_2_ + 2 HCO_3_^−^ + H^+^) in relation to H_2_ concentrations for samples from the methanic zones from AU2 to AU4. Gray horizontal bars indicate reaction Gibbs energies (G*_r_*) that are minimally exergonic, in a range between thermodynamic equilibrium (G*_r_* = 0 kJ mol^−1^ of reaction) and the proposed BEQ value (G*_r_* = −10 kJ mol^−1^ of reaction). H_2_ concentrations in which both reactions are exergonic are indicated by the areas between the dashed black lines. H_2_ concentrations with similar energy yields for both reactions, as would be expected under equal energies of both reactions, are indicated by the areas enclosed by dashed lines. Data points shown are the values for both reactions in Figure 8.

Things change in the underlying MZ, where aceticlastic methanogens (*Methanotrichaceae*) increase in numbers (Figure 7) despite Gibbs energies of aceticlastic methanogenesis that are barely exergonic (mostly −8 to −4 kJ mol^−1^) – even with measured methane concentrations that are underestimates due to outgassing during sampling (Figures 8A, 9A). These values suggest that aceticlastic methanogens are operating close to thermodynamic equilibrium at free energy yields lower than the BEQ (i.e., > −10 kJ mol^−1^). Alternatively, *Methanotrichacea* may not perform aceticlastic methanogenesis, but instead engage in CO_2_ reduction via DIET, as recently shown for *Methanothrix harundinacea* (Gao and Lu, 2021), a close relative of the *Methanotrichaceae* detected in our study (Figure 4). In the latter case, anaerobic acetate oxidation (reverse homoacetogenesis), which frequently occurs in methanogenic environments (Hattori, 2008; Dyksma et al., 2020), and was proposed to control acetate turnover in MZs further east in the Baltic Sea (Beulig et al., 2018), could also control acetate concentrations. Notably, our thermodynamic calculations suggest that at H_2_ concentrations of 10 nM, which may be required for methanogenic CO_2_ reduction with H_2_ to provide sufficient free energy for energy conservation (H^+^ translocation), anaerobic acetate oxidation is endergonic (Figure 8B). For syntrophic reactions involving anaerobic acetate oxidation to CO_2_ + H_2_ followed by CO_2_ reduction with H_2_ to both be exergonic and have similar Gibbs energies, a narrow window of H_2_ concentrations is necessary (AU2 and AU4: 1–2 nM; AU3: 1–4 nM; Figure 10). Within this window, Gibbs energies of both reactions provide free energy yields below BEQ values. Given that both pathways of acetate conversion to methane appear to only be minimally exergonic, our calculations cannot address which is more likely to be important in the MZs studied.

**Figure 11:**
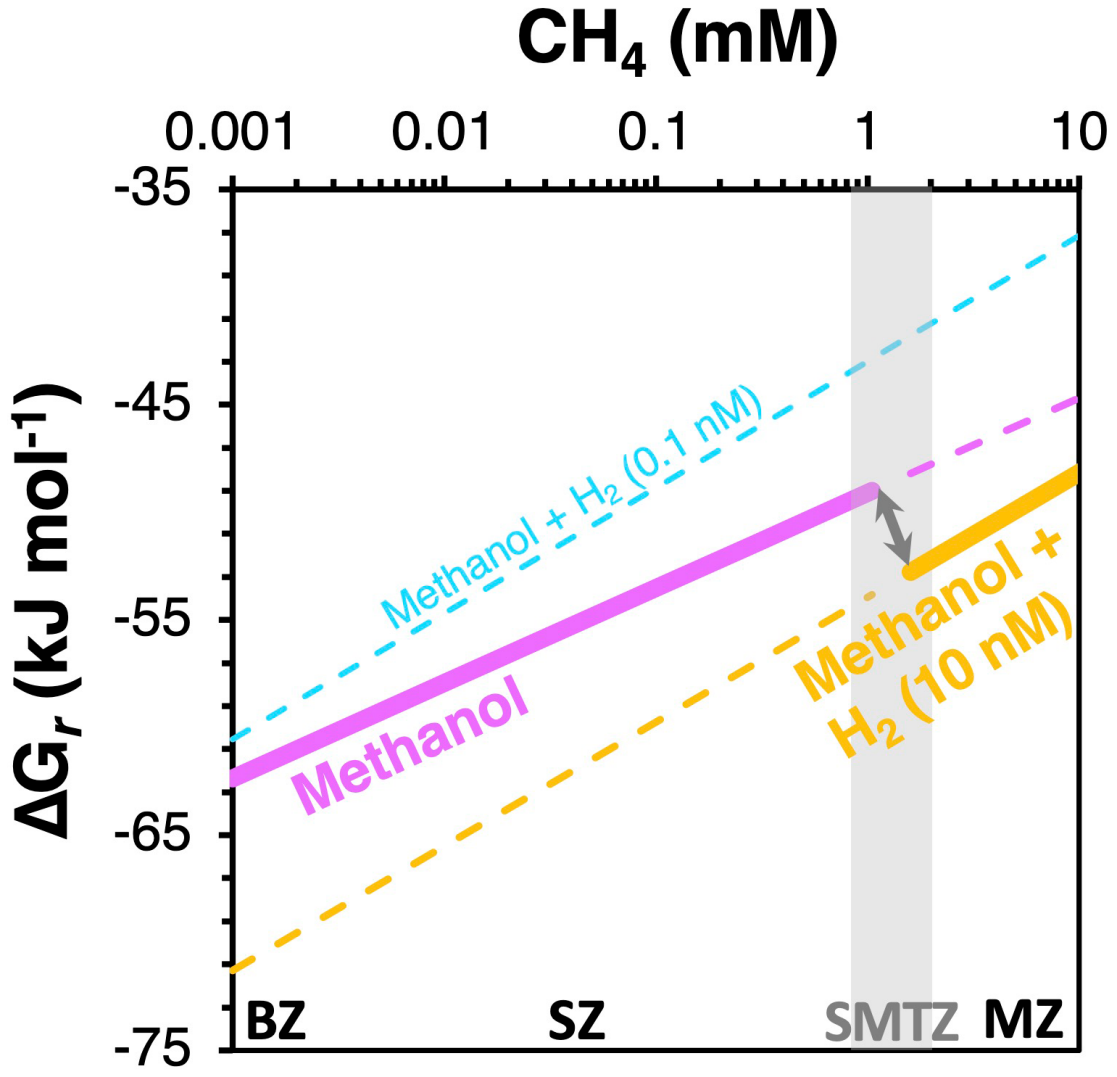
Proposed scenario for a switch in dominant methylotrophic pathway from methanol disproportionation (magenta) in the BZ and SZ to methanol reduction with H_2_ in the SMTZ and MZ (orange). We postulate that an increase in H_2_ concentrations from the BZ and SZ (cyan) to the MZ (orange) makes methanol reduction with H_2_ more energetically favorable than methanol disproportionation in the MZ and explains the observed vertical switch in dominant methylotrophic methanogens. Gibbs energies were calculated per mol of methanol. Calculations were done for two [H_2_] scenarios (0.1 nM, 10 nM). The shaded vertical bar indicates the SMTZ.

With respect to the observed shift from methyl disproportionation to methyl reduction as the dominant methylotrophic reaction from sulfate-rich to sulfate-depleted sediments, we explore the potential for *in situ* H_2_ concentrations to be the underlying driver (Figure 8C). This is, again, based on previous observations that H_2_ concentrations increase from metal and sulfate reduction-dominated sediments to methanic sediments (Lovley and Goodwin, 1988; Hoehler et al., 1998). Assuming that methanol is the substrate and present at 1 μM concentrations, both reactions are always highly exergonic in the sediments studied (−73 to −40 kJ mol^−1^ methanol). Yet, which reaction has higher free energy yields depends on H_2_ concentrations. Under “methanogenic H_2_ concentrations” (10 nM), methyl reduction always has higher free energy yields (more negative Gibbs energies) than methyl disproportionation. The opposite is the case at low H_2_ concentrations (0.1 nM), which are closer to values expected for sulfate and metal oxide reducing sediments. Even if H_2_ concentrations are at 1 nM, where Gibbs energy values are halfway between those for 10 nM and 0.1 nM on the x-axis of Figure 8C, methyl disproportionation yields higher free energy gains than methyl reduction. Our thermodynamic calculations thus offer an energetic explanation for the widely observed dominance of methyl disproportionating methanogens as the main methylotrophic methanogens in sulfate-rich surface sediments. By the same token, shifts to methyl reduction as the dominant methylotrophic pathway in deeper layers, which to our knowledge have never been documented, are potentially driven by higher H_2_ concentrations in deeper layers (for a conceptual diagram, see Figure 11).

## Conclusion

Our study indicates that methane-cycling archaeal communities in continental margin sediments generally follow two “axes of variation.” The first axis reflects the vertical biogeochemical zonation (i.e., BZ, SZ, SMTZ, and MZ), with sulfate concentrations as a likely key driver, while the second axis primarily reflects site-specific differences, e.g., in organic matter contents and reactivity. We, moreover, show that methane-cycling archaea undergo strong vertical shifts in community structure over time. As sediment horizons are buried to greater depths over time by seafloor sediment deposition, communities go through three successional stages – each characterized by different dominant taxa. The first successional stage is dominated by methyl disproportionating and specific CO_2_ reducing taxa (*BZ* & *SZ taxa*). These taxa decline in population size once they are buried to depths where methane concentrations become elevated (SMTZ). In these layers, *SMTZ taxa* that are largely linked to methanotrophic metabolism become dominant, showing clear evidence of net growth in response to increased methane availability. As sediment layers are buried further over time, to depths where sulfate concentrations become minimal, *SMTZ taxa* show population declines. In this methanic zone, a third successional stage emerges that is dominated by taxa involved in methanogenesis via CO_2_ and methyl group reduction. These *MZ taxa* already increase in population size once buried to the SMTZ – where sulfate concentrations become limiting and methanogenesis presumably becomes increasingly competitive with sulfate reduction for shared substrates – but only become dominant and reach their peak abundances once sulfate is depleted in the methanic zone. The three proposed successional stages are generally consistent with existing physiological knowledge on methane-cycling archaea and previous data on their distributions in marine sediments. Yet, the clear indication of net population growth based on vertical increases in gene copy numbers of major groups is novel. We propose that – following burial-related geochemical changes – periods of net population growth and proliferation of certain microbial taxa can occur in the cold sedimentary deep biosphere.

While the observed turnover and growth of specific methane-cycling archaeal taxa over time appears to be strongly influenced by sulfate concentrations and resulting competition of methanogens or cooperation of methanotrophs with sulfate reducers, our study also raises many new questions. Why do distinct taxa that share the same methanogenic pathway (e.g., CO_2_ reduction) or carbon substrate (methanol) dominate different successional stages? How is acetate converted to methane in methanogenesis zones? The answers to these questions are surprisingly unclear, despite decades of research on the marine sedimentary methane cycle. By outlining different thermodynamic scenarios, our study offers a theoretical framework for future investigations on the detailed mechanisms of methane-cycling in marine sediments.

## Data Availability

The datasets presented in this study can be found in online repositories. The names of the repository/repositories and accession number(s) can be found in the article/Supplementary material.

## Footnotes

^1^ http://www.bioinformatics.babraham.ac.uk/projects/fastqc

^2^ https://github.com/lh3/seqtk

^3^ www.arb-home.de

^4^ https://drive.google.com/drive/folders/1G8GeJuYsIX4MLv5-LaUQHD9f9F9rfIAu

^5^ http://www.R-project.org
