# Supplementary material for: Drivers of methane-cycling archaeal abundances, community structure, and catabolic pathways in continental margin sediments
Source: Front Microbiol. 2025 Feb 6;16:1550762. doi: 10.3389/fmicb.2025.1550762 (PMC11840676; doi:10.3389/fmicb.2025.1550762)
Supplement: Supplementary file 2 [file Data_Sheet_2.docx]

**Supplementary Information**

**Table S1.** Thermodynamic data of aqueous educts and products under standard conditions.

| **Chemical species** | **∆G*_f_*^°^**  **(kJ mol^-1^)** | **∆H*_f_*°**  **(kJ mol^-1^)** | **∆V*_f_*°**  **(cm^3^ mol^-1^)** | **Source** |
| --- | --- | --- | --- | --- |
| H^+^ | 0.0 | 0.0 | 0.0 | Shock et al. (1997) |
| H_2_ | 17.6 | -4.2 | 25.2 | Wagman et al. (1982), Shock and Helgeson (1990) |
| water | -237.2 | -285.8 | 18.0 | Amend and Shock (2001) |
| bicarbonate | -586.9 | -692.0 | 24.6 | Wagman et al. (1982), Shock et al. (1997) |
| formate | -351.0 | -425.7 | 26.2 | Shock and Helgeson (1990) |
| acetate | -369.4 | -486.4 | 40.5 | Shock and Helgeson (1990) |
| methanol | -175.4 | -246.5 | 38.2 | Shock and Helgeson (1990) |
| sulfate | -744.96 | -910.21 | 13.88 | Shock et al. (1997) |
| sulfide | 11.97 | -16.12 | 20.65 | Shock et al. (1997) |

**Figure S1.** Correlation heatmap to examine the relationships between absolute gene abundances of dominant *mcr*A groups and environmental variables, and between dominant *mcr*A groups. Αll correlations are with *p*<0.05.

**Figure S2.** Correlation heatmap to examine relationships between absolute and relative gene abundances of dominant *mcr*A groups (this study) with absolute and relative gene abundances of dominant microbial groups based on 16S rRNA gene sequence data (from Deng et al., 2020). Αll correlations are with *p*<0.01.

**Supplementary References**

Amend, J. P., and Shock, E. L. (2001). Energetics of overall metabolic reactions of thermophilic and hyperthermophilic archaea and bacteria. *FEMS Microbiol. Rev.* 25, 175-243.

Shock, E. L., and Helgeson, H. C. (1990). Calculation of the thermodynamic and transport properties of aqueous species at high pressures and temperatures: standard partial molal properties of organic species. *Geochim. Cosmochim. Acta* 54, 915-945.

Shock, E. L., Sassani DC, and Willis M, Sverjensky DA. (1997). Inorganic species in geologic fluids: correlations among standard molal thermodynamic properties of aqueous ions and hydroxide complexes. *Geochim. Cosmochim. Acta* 61, 907-950.

Wagman, D. D., Evans, W. H., Parker, V. B., Schumm, R. H., Halow, I., Bailey, S. M., Churney, K. L., and Nuttall, R. L. (1982). The NBS tables of chemical thermodynamic properties: Selected values for inorganic and C1 and C2 organic substances in SI units: *J. Phys. Chem. Ref. Data*, 11, 392 p.
